# Genotypes of *Eruca vesicaria* subsp. *sativa* grown in contrasting field environments differ on transcriptomic and metabolomic levels, significantly impacting nutritional quality

**DOI:** 10.3389/fpls.2023.1218984

**Published:** 2023-11-02

**Authors:** Luke Bell, Martin Chadwick, Manik Puranik, Jake Jasper, Richard Tudor, Lisa Methven, Carol Wagstaff

**Affiliations:** ^1^ School of Agriculture, Policy & Development, Crop Sciences, University of Reading, Reading, United Kingdom; ^2^ School of Chemistry, Food & Pharmacy, Food & Nutritional Sciences, University of Reading, Reading, United Kingdom; ^3^ Vegetable Plant Breeding, Elsoms Seeds Ltd., Spalding, United Kingdom

**Keywords:** glucosinolates, isothiocyanates, brassicaceae, transcriptome sequencing, volatile organic compounds, arugula

## Abstract

Rocket (*Eruca vesicaria* subsp. *sativa*) is a source of sulfur-containing glucosinolates (GSLs). GSLs and their breakdown hydrolysis products (GHPs) are responsible for health-related benefits, such as anti-cancer and anti-neurodegenerative properties. Understanding how phytochemical composition changes between cultivation environments is key to developing cultivars with improved nutritional quality. Two consecutive harvests (first and second regrowth) of crops, grown in both Italy and the UK, were used to determine the phytochemical and transcriptomic differences between six lines of *Eruca*. Samples were taken upon delivery from field sites (D0) and after five days of cold storage (D5) for each location. Leaves were analysed for sulfur content, volatile organic compounds (VOCs), GSLs, GHPs, and sugars. Transcriptome data were associated with metabolite profiles to identify differentially expressed genes between plants grown in the two environments. VOC compounds (carbon disulfide, methyl thiocyanate) were associated with growth environment and with differences in sulfur metabolism gene expression (*APR2, LSU2, LSU3, SDI1, SiR*), GSL biosynthesis (*MYB28, FMOGS-OX2*) and GHP formation (*ESM1, TGG1, TGG2*). The concentrations of sugars were an order of magnitude greater in UK grown samples (up to 29.9 mg g^-1^ dry weight; dw). Sulfur content was significantly higher in the Italy plant samples (11.4 – 20.1 mg g^-1^ dw), which was in turn associated with higher concentrations of GSLs (pentyl GSL, up to 15.8 μmol g^-1^ dw; sinigrin, up to 0.005 μmol g^-1^ dw; glucoraphanin, up to 5.1 μmol g^-1^ dw; glucorucolamine, up to 23.6 μmol g^-1^ dw; neoglucobrassicin, up to 5.3 μmol g^-1^ dw) and hydrolysis products (sativin, up to 13.5 μmol g^-1^ dw; erucin, up to 1 μmol g^-1^ dw; sulforaphane, up to 34.7 μmol g^-1^ dw). VOC profiles of plants cultivated in the UK were distinct from Italy grown plants, with higher relative abundances of alkanes and esters in second cut and shelf-life (D5) samples. The data indicate a significant interaction of cultivar response with environment, highlighting the difficulty of producing *Eruca* crops with consistent phytochemical and postharvest traits. Genes with differential expression between plants grown in Italy and the UK could be used as markers of phytochemical quality and composition.

## Introduction

‘Salad’ rocket (*Eruca vesicaria* subsp. *sativa*; also known as arugula and rucola) is a member of the Brassicaceae family and is closely related to thale cress (*Arabidopsis thaliana*), cabbage (*Brassica oleracea*), and radish (*Raphanus sativus*; Bell et al., 2020a). It is widely consumed across the world as a leafy vegetable and has become naturalized on every inhabited continent (Tripodi et al., 2021). Leaves are known for their characteristic peppery flavour, pungent aroma, and bitter taste (Bell et al., 2017a); however, the intensity of these attributes is closely linked with the cultivation environment (Bell et al., 2020b). Previous research has shown that high growth temperatures are associated with increased concentrations of defensive phytochemicals called glucosinolates (GSLs) (Jasper et al., 2020). These compounds are hydrolysed by endogenous myrosinase enzymes and their cofactors to produce volatile organic chemical (VOC) hydrolysis products such as isothiocyanates (ITCs; Blažević et al., 2019). ITCs compounds are associated with beneficial health effects in humans (Bell and Wagstaff, 2017), but some impart bitterness and pungency that a large proportion of consumers find repellent (Bell et al., 2017b; Oloyede et al., 2021). Previous research on the sensory properties and consumer acceptability of *Eruca* have found that most consumers prefer sweet, peppery leaves with low levels of pungency and bitterness (Bell et al., 2017b). Given that these attributes are responsive to cultivation practices and growth conditions it is challenging to produce crops with consistent quality between growing regions and across growing seasons.

GSL profiles of *Eruca* plants are now well established (Bell et al., 2021), however the impacts of environment on the formation and abundance of their hydrolysis products (GHPs) are less well studied. There are many theoretical ways in which GSL hydrolysis can be promoted or inhibited depending on the native cellular environment (pH and temperature; Nastruzzi et al., 2000), presence/absence of enzyme co-factors (Backenköhler et al., 2018) and abundance of other cellular metabolites (including sugars, such as glucose, fructose, galactose, and sucrose; Román et al., 2018). Few studies have examined how different field conditions impact the formation of GHPs from *Eruca* leaves, which is problematic, as the generation of these compounds underpins the basis of sensory properties and postharvest quality traits. The practice of harvesting *Eruca* plants multiple times also presents challenges in maintaining the consistency of produce, as increases in GHPs make the leaves more pungent (Bell et al., 2020b). The genetic mechanisms regulating hydrolysis are poorly understood, and evidence from previous studies in related species suggests that there is a significant genotypic component that determines the abundance of GHPs (Witzel et al., 2019) and other VOCs (Guijarro-Real et al., 2020) that are produced.

Previous research has identified sugars as a component that reduce or ‘mask’ the pungency and bitterness of *Eruca* leaves (Bell et al., 2017a). The accumulation of sugars in leaves is closely linked with environment (Song et al., 2017), however the regulation of sugar metabolism in response to environmental factors remains unclear. *Eruca* itself is a Mediterranean species and has become naturalized in colder regions of the world, leading researchers to hypothesize that the higher concentrations of sugars observed in the UK may be due to a cold stress response (Bell et al., 2022). As demonstrated by Román et al. (2018), free sugars themselves may also act as inhibitors of myrosinase, blocking GSL hydrolysis and reducing the abundance of GHPs produced. Understanding the genes associated with sugar accumulation in leaves will assist breeders in selecting lines which have tempered pungency (which are favored by a many consumers), but which also retain high concentrations of secondary metabolites (GSLs) and produce health-related breakdown products (GHPs).

In this study we conducted metabolomic and transcriptomic profiling of six *Eruca* lines. These lines were of high (68, 112, 130) and low (21, 25, 72) GSL content and were grown in Italy and the United Kingdom. Leaves of each line were sampled at day 0 (D0; intake of samples at the University of Reading) and day five of shelf-life (D5; cold storage at 4°C) for both first and second harvests of plants. Leaves of the six lines underwent transcriptome sequencing (RNAseq) at two consecutive harvest time points and their corresponding shelf life time point (D5) for both the Italy and UK trials. We hypothesised that specific phytochemical (such as GSLs and sugars), VOC abundances, and GHPs, would be associated with differential expression patterns of genes involved with sulfur assimilation, GSL biosynthesis and hydrolysis, sugar metabolism, and abiotic stress response pathways.

Based on previous field trial observations (Bell et al., 2022) we also hypothesised that UK-grown plants would contain significantly higher concentrations of sugars compared with Italy-grown. The rationale for this hypothesis was based upon reports from growers and processors that rocket leaves produced in the UK are typically of a lower quality (lacking pungency and postharvest longevity) than those from Italy owing to suboptimal pre-harvest temperatures and humidity; this in turn leads to greater levels of disease and stress burden, increasing respiration and the increased need for sugars in primary metabolism (Sami et al., 2016). Previous controlled environment transcriptome and phytochemical analyses of parent line material (of which the plants in this study are derived) has also highlighted the effect of genotype that underpins differences in phytochemical and nutritional traits of *Eruca*. In this study we aimed to determine if such genetic factors were consistent between growing locations in terms of the relative abundances of GSLs and GHPs produced by lines in each environment (Italy and the UK). Our goal in this research is to study and understand *Eruca* lines from the perspective of driving high nutritional and phytochemical yield for the improvement of consumers’ long-term health and acceptance.

## Materials and methods

### Plant materials

Six lines of ‘salad’ rocket were selected from a segregating F_2_ mapping population of 139 lines based on their high and low abundances of GSLs across two growing locations (Bell et al., 2022). The selected lines were referred to as 21 (low GSL), 25 (low GSL), 68 (high GSL), 72 (low GSL), 112 (high GSL), and 130 (high GSL). Twenty plants of each line were grown, and bulk pollinated (F_4_B_1_) in cages within a glasshouse environment at Elsoms Seeds Ltd. (Spalding, UK) to produce sufficient seed amounts for commercial scale productions.

The selected lines were grown in two contrasting environments reflective of commercially produced ‘salad’ rocket leaves: a polytunnel near Rome, Italy (41°55’31.1”N 12°08’15.8”E) in September 2018, and in an open field near Owermoigne, Dorchester, UK (50°40’40.9”N 2°19’34.3”W) in July 2019. The dates were selected because they are representative of the peak output of rocket crops in the two respective growing locations. The UK season is substantially shorter than that in Italy and so production peaks earlier in the year and coincides with increased summer demand for domestic salad leaves. The Italian season peaks later when the UK-domestic season is beginning to decline. For both trials seeds were broadcast seeded in pre-prepared parallel soil beds, as per standard commercial practices in the respective countries. See **Table 1** for the weather data of each respective trial.

**Table 1:** Average daily weather data for two ‘salad’ rocket field trials, in Italy and the United Kingdom.

Trial location & harvest number	Average Temp (°C)	Temp max (°C)	Temp min (°C)	Rainfall (mm)	Average no. daylight hours received	Cloud (%)	Humidity (%)
Italy 1st harvest	23.1	25.5	20.7	n/a*	12.0	22.7	61.4
Italy 2nd harvest	20.8	23.0	19.4	n/a*		29.8	69.1
UK 1st harvest	16.5	18.3	14.2	19.5	15.9	35.5	82.4
UK 2nd harvest	16.9	18.5	14.8	10.3		50.4	83.2

* Italy trial grown in a polytunnel with daily overhead irrigation.

n/a, not applicable.

First harvest of Italy grown plants was 23 days after sowing (DAS), and a second harvest at 30 DAS. The UK trial first cut was taken 26 DAS, and a second cut at 37 DAS. The difference in harvest times between locations was due to the difference in respective growth rates, with the Italy trial establishing and re-growing more quickly than the UK trial. Both trials were bordered by a commercial guard crop of commercial salad rocket. Both trials were harvested by band blade machines in the morning. Leaves were placed into crates and vacuum cooled using on-farm facilities at each respective site. Leaves from the Italy trial were transported to the UK in a temperature-controlled van (4°C) and delivered to a processing facility in Owermoigne, Dorchester, UK. Leaves from the UK trial were stored for two days postharvest in a 4°C cold store at the same site to match the duration of leaves in transit from Italy. Each of the rocket lines were washed, rinsed, and spin-dried by hand according to the protocol of Jasper et al. (2020). A sample of 50 g of leaves were taken randomly from crates of each line, placed into microperforated plastic bags, and heat sealed (*n* = 12 bags per line, per trial). Each 50 g bag constituted a representative biological sample for the subsequent analyses. The sealed bags were then transported via temperature-controlled van (4°C) to the University of Reading School of Chemistry, Food & Pharmacy. All bags were stored overnight in a cold store at 4°C.

Six bags of each line were used for Solid Phase Microextraction (SPME) of VOCs by Gas Chromatography Mass Spectrometry (GC-MS; fresh leaves), and six for other laboratory analyses (lyophilized; see following sections). Three bags were used as D0 samples and the remaining three were stored for a period of five days at 4°C and tested constituting the D5 shelf-life samples. This replicates a typical period that bagged rocket leaves may spend in a domestic refrigerator (Jasper et al., 2021). The six bags for sulfur, GSL, GHP, and sugar extractions were divided in the same fashion and stored for the same corresponding time points. These samples were frozen on dry ice at -80°C to preserve phytochemical (and RNA; see following section) integrity. Samples of each line therefore consisted of the following: 1^st^ cut D0, 1^st^ cut D5, 2^nd^ cut D0, and 2^nd^ cut D5 for both the Italy and UK field trials (see **Supplementary File S1** for sampling plan).

### Solid Phase Microextraction of VOCs and GC-MS analysis

A 2 g sample of fresh leaves for each cultivar was taken in triplicate from sealed bags. The method of extraction and analysis by SPME GC-MS was identical to that presented by Bell et al. (2021). A list of detected compounds from both Italy and UK trials is presented in **Supplementary File S2**.

### Phytochemical and elemental sulfur extraction and analysis

Bags of each line used for metabolite analyses were lyophilized in batches for three days. Dried leaves were ground into a fine powder using a Wiley Mini Mill (Thomas Scientific, Swedesboro, NJ, USA).

Intact GSLs were extracted and analysed by Ultra High Pressure Liquid Chromatography Triple Quadrupole Mass Spectrometry (UHPLC-MS/MS) according to the method presented by Bell et al. (2021). Authentic standards were purchased from PhytoPlan (Heidelberg, Germany): glucoiberin (GIB; 99.61%), progoitrin (PRO; 99.07%), sinigrin (SIN; 99%), glucoraphanin (GRA; 99.86%), glucoalyssin (GAL; 98.8%), gluconapin (GNP; 98.66%), 4-hydroxyglucobrassicin (4HGB; 96.19%), glucotropaeolin (GTP; 99.61%), glucoerucin (GER; 99.68%), glucobrassicin (GBC; 99.38%), gluconasturtiin (GNT; 98.38%). All compound purities were determined by High Performance Liquid Chromatography Diode Array Detector (HPLC-DAD). Pentyl GSL (PEN), glucorucolamine (GRM), glucoputranjivin (GPJ), diglucothiobeinin (DGTB), glucoberteroin (GBT), glucosativin (GSV), dimeric 4-mercaptobutyl GSL (DMB), 4-methylpentyl GSL (4MP), hexyl GSL (HEX), and butyl GSL (BUT) were semi-quantified using SIN, and 4-methoxyglucobrassicin (4MGB) and neoglucobrassicin (NGB) were semi-quantified using the GBC standard, as no authentic standards were available. GLSs were identified by matching ion spectra and fragmentation with standards and literature sources (Cataldi et al., 2007; Rochfort et al., 2008; Agneta et al., 2012; Maldini et al., 2017; Shi et al., 2017; Andini et al., 2019). PEN, GRM, GPJ, 4MP, and BUT are tentatively identified due to the lack of robust literature ion data and the possibility of being isomers of GSLs with identical mass (**Table 2**).

**Table 2:** Glucosinolate compounds identified in *Eruca vesicaria* subsp. *sativa* extreme lines by UPLC-MS/MS.

Glucosinolate	Common name	Abbreviation	Rt	Precursor ion ([M-H]-)	Quantitative transition	Qualitative transition
Methylsulfinylpropyl	Glucoiberin ^*^	GIB	1.061	422	422>357	422>97
Pentyl ^$a^	–	PEN	1.08	388	388>75	388>97
(*R*)-2-hydroxy-3-butenyl	Progoitrin ^*^	PRO	1.336	388	388>74	388>97
Allyl	Sinigrin ^*^	SIN	1.523	358	358>258	358>97
4-methylsulfinylbutyl	Glucoraphanin ^*^	GRA	1.598	436	436>371	436>97
4-(cystein-*S*-yl)butyl	Glucorucolamine ^$a^	GRM	1.654	494	494>406	494>217
5-(methylsulfinyl)pentyl	Glucoalyssin ^*^	GAL	2.421	450	450>206	450>97
1-methylethyl	Glucoputranjivin ^$a^	GPJ	2.447	360	360>75	360>97
3-butenyl	Gluconapin ^*^	GNP	2.489	372	372>258	372>97
4-(β-D-glucopyranosyldisulfanyl)butyl	Diglucothiobeinin ^a^	DGTB	2.501	600	600>290	600>97
5-(methylthio)pentyl	Glucoberteroin ^$a^	GBT	2.593	434	434>95	434>97
4-hydroxy-3-indolylmethyl	4-hydroxyglucobrassicin ^*^	4HGB	2.619	463	463>285	463>97
4-mercaptobutyl	Glucosativin ^a^	GSV	2.684	406	406>74	406>97
Dimeric 4-mercaptobutyl ^a^	–	DMB	2.839	811	405>80	405>97
Benzyl	Glucotropaeolin ^*^	GTP	2.879	408	408>259	408>97
4-methylthiobutyl	Glucoerucin ^*^	GER	2.919	420	420>74	420>96
Indolyl-3-methyl	Glucobrassicin ^*^	GBC	3.102	447	447>259	447>97
4-methoxyindolyl-3-methyl	4-methoxyglucobrassicin ^b^	4MGB	3.173	477	477>75	477>97
2-phenethyl	Gluconasturtiin ^*^	GNT	3.419	422	422>259	422>97
1-methoxyindolyl-3-methyl	Neoglucobrassicin ^b^	NGB	3.526	477	477>75	477>97
4-methylpentyl ^$a^	–	4MP	3.695	402	402>259	402>97
Hexyl ^$a^	–	HEX	3.726	402	402>119	402>97
Butyl ^$a^	–	BUT	4.176	375	375>256	375>180

^*^ Authentic standard; ^a^ semi-quantified using sinigrin; ^b^ semi-quantified using glucobrassicin; ^$^ tentative identification.

The “-” denotes that there is no common name for the compound.

Semi-volatile GHPs (sativin, SAT; erucin, ERU; sulforaphane, SF; and bis(4-isothiocyanatobutyl) disulfide, B4IBD) and elemental sulfur compositions of *Eruca* leaves were extracted and analysed in identical fashion to Bell et al. (2020a). GHPs were semi-quantified by GC-MS using authentic SF (Merck-Sigma, Gillingham, UK) as an external standard. Sulfur content was determined by Inductively Coupled Plasma Optical Emission Spectroscopy (ICP-OES) using the radial signal at 181.975 nm.

Sugars were quantified using authentic standards (Merck-Sigma) by High Pressure Ion Chromatography Mass spectrometry (HPIC-MS). The method of extraction was identical to that of Bell et al. (2020a) with the following modification: 1.5 mL aliquots of rocket leaf extracts were diluted with 1.5 mL of dH_2_O containing an internal standard of rhamnose (20 mM) to give a final concentration of 10 mM. Samples were analysed using a Dionex ICS 6000 (ThermoFisher Scientific, Loughborough, UK) with an eluent gradient composed of 10 mM NaOH (A) and 250 mM NaOH (B). Gradient timetable was as follows: (i) 0 min (A–B, 100:0, v/v); (ii) 0–35 min (A–B, 100:0, v/v); (iii) 35–40 min (A–B, 0:100, v/v); (iv) 40–50 min (A–B, 0:100, v/v); (v) 50–55 min (A–B, 100:0, v/v). The flow rate was 1 mL/min, with a column temperature of 20°C.

### RNA extraction, transcriptome sequencing, bioinformatics, and co-expression analysis

RNA extraction, sequencing, bioinformatics and co-expression analysis were performed according to the methods described by Bell et al. (2020a). Extracts were taken from homogenized lyophilized samples corresponding to the same samples and time points outlined for the chemical analyses (**Supplementary File S1**). All samples were checked for degradation and contamination by gel electrophoresis, Qubit fluorometer, NanoDrop (Thermo Fisher Scientific, Loughborough, UK) and Agilent 2100 Bioanalyzer (Agilent, Santa Clara, CA, USA). Samples not meeting all quality criteria were omitted from downstream analyses (UK trial, lines 21, 25, and 68; 2^nd^ cut D5). RNA library preparation, sequencing, and bioinformatics were performed by Novogene Ltd. (Cambridge, UK) as a service. Full gene expression and annotation data of all samples can be found in the University of Reading Research Data Archive (https://doi.org/10.17864/1947.000458). A co-expression analysis of expression data was performed using the webCEMiTool (Co-Expression Module Identification Tool) pipeline (Cardozo et al., 2019) according to the parameters described by Bell et al. (2020a). Heatmaps and volcano plots were generated using NovoSmart software (Novogene Ltd.).

### Statistical analysis

Phytochemical, VOC, and elemental data sets were analysed using XL Stat (Addinsoft, Paris, France). Data were tested for outliers (Grubb’s test) and these values were removed from downstream analyses. Shapiro-Wilk normality tests were conducted for each variable and concluded to fit a normal distribution. Analysis of variance (ANOVA) tests were conducted with line, geographic region (country), harvesting points and storage time, as treatment effects (with interactions); after which *post hoc* Tukey’s Honest Significant Difference (HSD) was used for pairwise comparison tests (p<0.05). Summary data of these analyses are presented in **Supplementary File S3**.

Principal Component Analysis (PCA) with Pearson’s correlation analysis (*n*-1) was performed on sulfur content, phytochemical (GSL, GHP, and sugar) concentrations, and VOC abundances to determine if samples separated according to cultivation location (Italy and UK). A separate PCA analysis of Italy sample data was conducted in conjunction with 258 genes were selected based on genome annotation data (Bell et al., 2020a) and their putative identifications. These included genes associated with sulfur metabolism, GSL biosynthesis, GSL hydrolysis, cell redox homeostasis, GSL transport, defense response, VOC synthesis, and sugar metabolism. This was done to elucidate differences between genotypes and understand the associations between gene expression and metabolite abundance.

## Results

### Sugar composition

Phytochemical constituent data and statistical results are collated in **Supplementary File S4**. Sugar analysis of leaves showed a significant difference in concentrations between Italy and UK-grown plants (*p* = <0.001). The most abundant sugar in Italy samples was fructose, whereas in the UK grown plants glucose was the major sugar component. Total concentrations of sugars in D0 samples were an order of magnitude greater in UK plants than those form Italy (**Supplementary File S4**). The highest concentration observed was in line 112 (UK, 1^st^ cut, D0), 29.9 mg g^-1^ dw. This compares with only 0.2 mg g^-1^ dw for the corresponding Italy-grown plant sample. This difference in sugar concentrations is consistent with previous observations (Bell et al., 2022).

### Sulfur content

**Figure 1:**
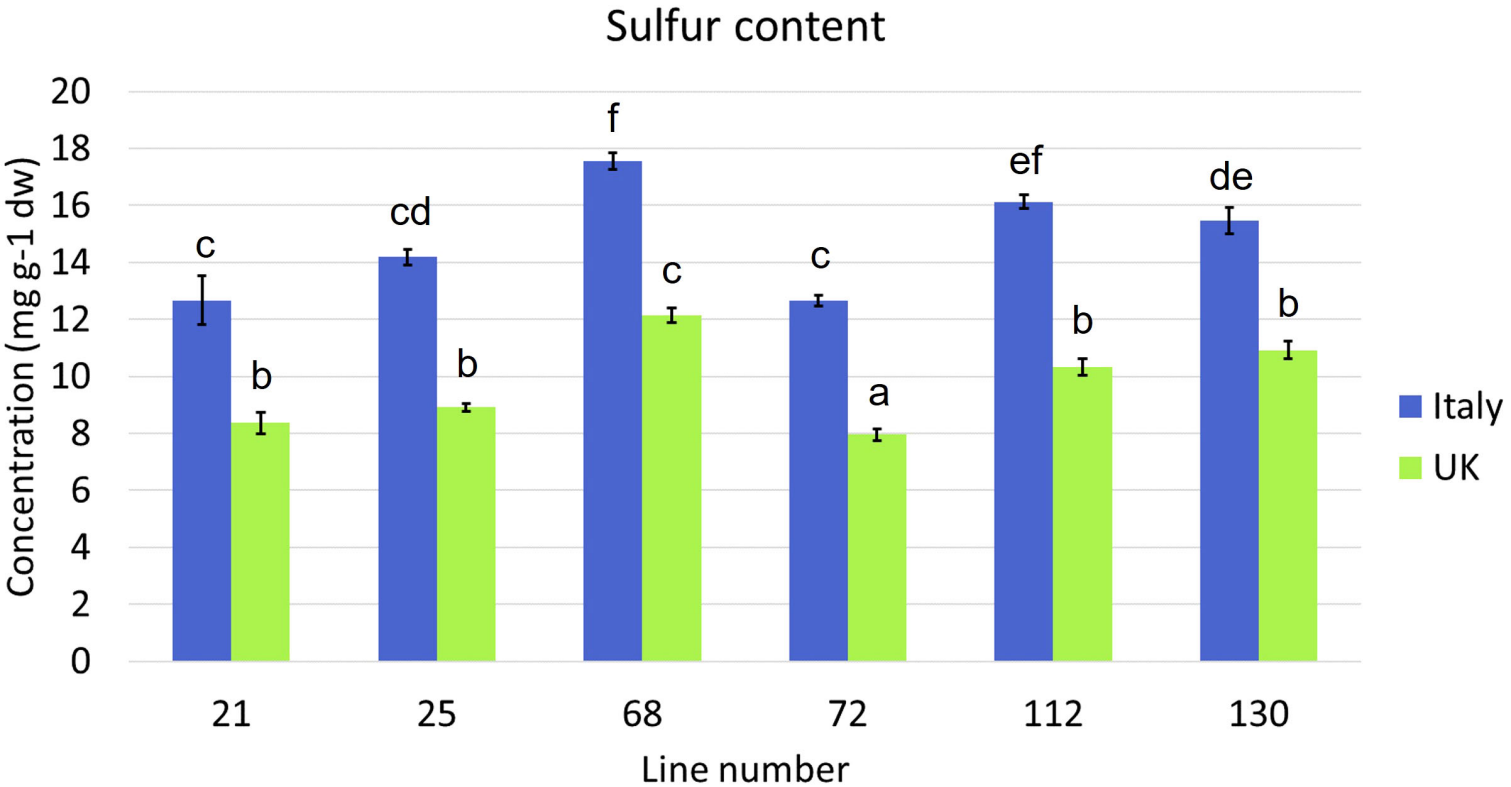
Average sulfur content of six *Eruca vesicaria* subsp. *sativa* extreme lines grown in Italy and the UK. Error bars represent standard error of the mean. Letters above bars indicate significant differences observed for sulfur content for each cultivar between growing locations (*p* = <0.05).

Sulfur content was significantly higher in Italy plants than in UK plants (*p* = <0.0001), with the highest average amounts observed in line 68 (Italy, 17.6 mg g^-1^ dw; UK, 12.1 mg g^-1^ dw; **Figure 1**). The lowest average concentrations were found in line 72 in each trial (Italy, 12.6 mg g^-1^ dw; UK, 8.0 mg g^-1^ dw).

### Volatile organic chemical composition

**Figure 2:**
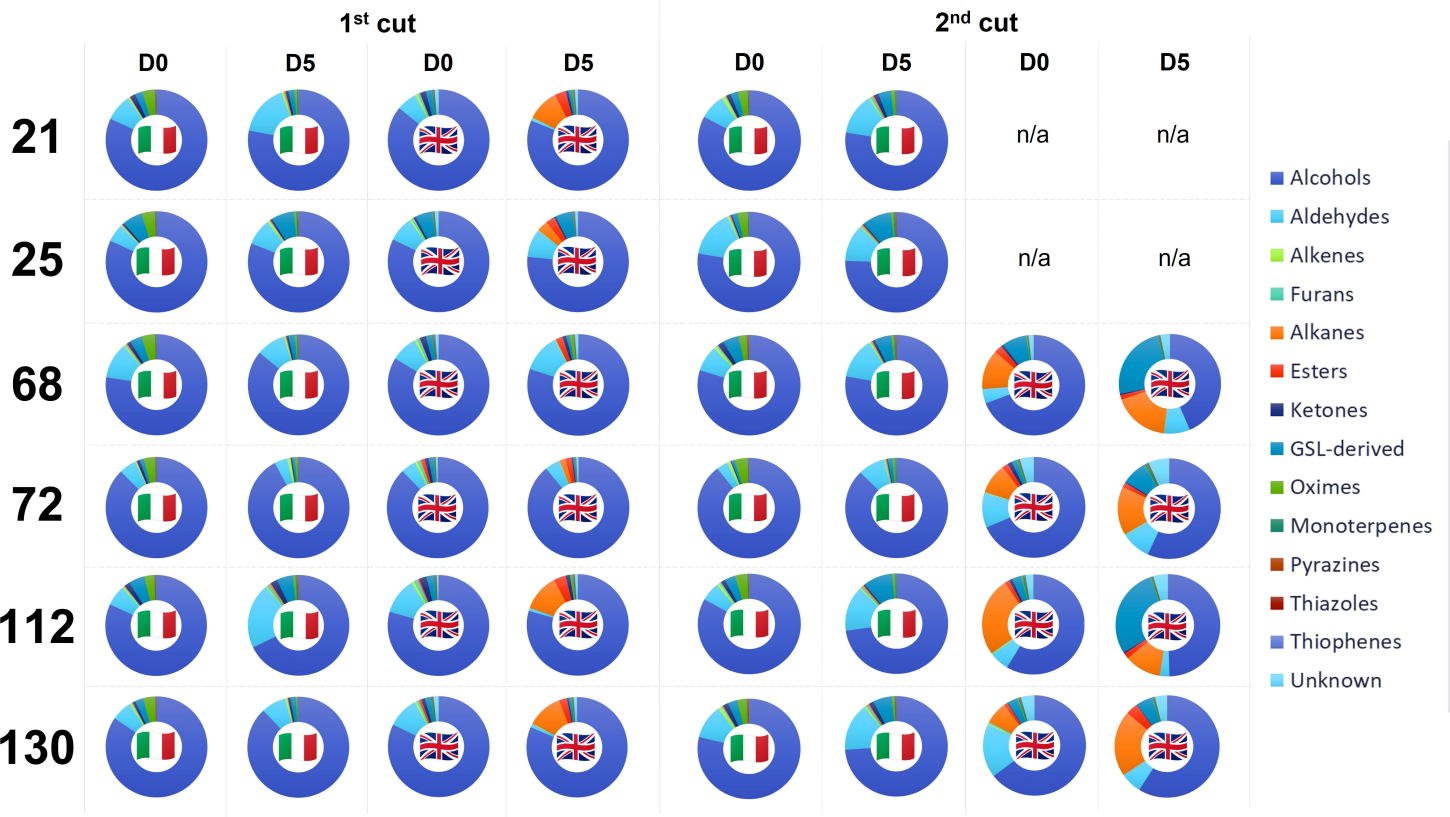
Volatile organic compound (VOC) relative percentage composition for six *Eruca* extreme lines grown in Italy and the UK across two cuts (1^st^ and 2^nd^) and two time points; intake (D0) and after five days of shelf-life storage (D5). See inset for VOC colour coding. N.B. UK material for lines 21 and 25 at 2^nd^ cut were not of sufficient quality for robust analysis. See inset for colour coding, which proceeds clockwise from 0°. n/a, not applicable.

VOC abundances are presented in **Figure 2**. A full list of detected VOCs, their mean (normalized) values, and the significance of treatment effects is presented in **Supplementary File S2** and **Supplementary File S3**. The 1^st^ cut D0 VOC profiles between the two trials are similar, however compositional differences are apparent in D5 samples. While Italy plant profiles remained consistent, there is an evident shift in VOC relative abundances in UK leaves with increases in alkanes and esters in all lines (except for 68). In 2^nd^ cut samples this difference is more pronounced, with relative percentage increases in alkanes, esters, and GSL-derived compounds, with corresponding reduction in the abundance of alcohols (**Supplementary File S3**).

96 VOC compounds were identified (38) or tentatively identified (58) across the two cultivation locations and six genotypes tested. Only 31 compounds were detected in both Italy and UK samples (**Supplementary File S2**), and of these, significant differences in relative abundance were observed for 25 compounds (*p* = <0.05; **Supplementary File S3**). A high level of significance was observed for interacting factors of specific VOC relative abundances. 3-methylfuran was primarily observed in Italy-grown samples, with abundances significantly associated with the cultivar x shelf life (time) interaction (*p* = <0.001). In UK grown samples, the interaction between harvest time and shelf life (time) was significant for several compounds, such as (2*Z*)-pentene, methyl sulfide, 3-methylfuran, carbon disulfide, (3*Z*)-3-hexen-1-ol, and β-cyclocitral (*p* = <0.001). Methyl thiocyanate (a potential breakdown product of ITCs) varied significantly for the cultivar x country interaction effect (*p* = <0.001), indicating a strong genotype x environment (GxE) effect in determining its abundance. It has been linked with sulfurous and onion-like aromas (**Supplementary File S2**). Carbon disulfide, 3-methylfuran, and methyl thiocyanate abundances also produced a significant cut x country interaction effect (*p* = <0.001), and methyl sulfide, (3*E*)-1,3-pentadiene, 3-methylfuran, and 5-(methylsulfanyl)pentanenitrile abundances had strong interacting effects between shelf life (time) and country. 5-(methylsulfanyl)pentanenitrile is of particular interest as a hydrolysis product of GBT, which has been described as having a radish, cabbage, and broccoli-like aroma (Bell et al., 2021; **Supplementary File S2**).

### Glucosinolate composition

**Figure 3:**
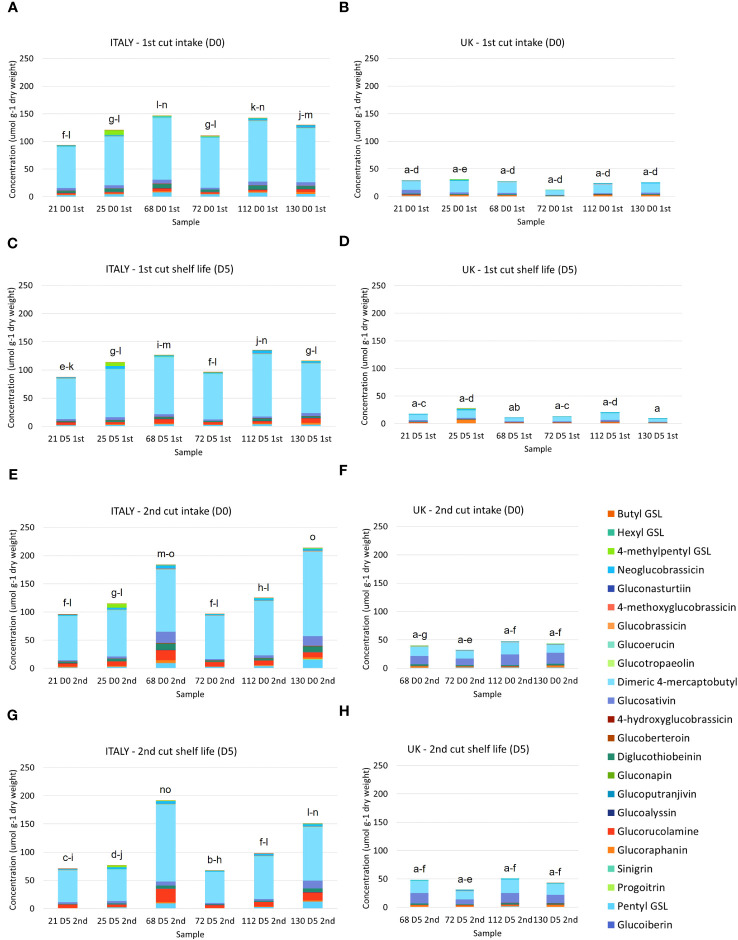
Glucosinolate concentrations of six *Eruca vesicaria* subsp. *sativa* extreme lines grown in Italy and the UK. Letters above bars indicate significant differences observed for total glucosinolate concentrations between each sample and environment. **(A)** Italy, first cut intake (D0); **(B)** UK, first cut intake (D0); **(C)** Italy, first cut shelf life (D5); **(D)** UK, 1st cut shelf life (D5); **(E)** Italy, second cut intake (D0); **(F)** UK, second cut intake (D0); **(G)** Italy, second cut shelf life (D0); **(H)** UK, second cut shelf life (D5).

23 GSL compounds were identified in *Eruca* samples in agreement with previous observations (Bell et al., 2021; **Table 2**). Concentrations of GSLs were significantly higher in Italy samples compared with UK. Italy 2^nd^ harvest (D0) contained the highest average concentrations (lines 68 and 130 – 191.9 μmol g^-1^ DW, 213.5 μmol g^-1^ DW, respectively; **Figure 3E**). Line 72 produced the lowest average concentrations across both the Italy and UK trials (67.9 μmol g^-1^ DW, Italy 2^nd^ cut D5, and 11.9 μmol g^-1^ DW, UK 1^st^ cut D0, respectively). GSL profiles of Italy-grown plants were dominated by DMB, with relatively high concentrations of PEN, GRM, DGTB, and GSV. Of note in line 25 were high concentrations of the tentatively identified 4MP (up to 8.7 μmol g^-1^ DW, Italy 1^st^ cut D0), which was only observed in very low concentrations in other lines. UK lines contained a lower abundance of DMB relative to GSV, with a significant GxE interaction observed for DMB (*p* = <0.01) as well as total GSL concentrations (*p* = <0.001; **Supplementary File S3**.

Of note was the presence of PEN, GBT, and GTP in *Eruca* samples, which are rarely reported as constituents of the species GSL profile. The abundances reported here are substantial, and in the case of GBT and GTP verified by comparison with authentic standards. PEN was previously tentatively reported by Bell et al. (2021) in *Eruca* and hypothesised to have been confused with PRO (both *m/z* 388) by other studies, and which has been regularly reported. Our analysis showed that the PRO standard (and epiprogoitrin, not found in *Eruca* but available as a standard) eluted later than the peak identified as PEN. The data suggest that GSL analyses should be conducted with greater attention to such details, and that more comprehensive screening of samples for compounds is required to avoid underreporting of important GSL constituents.

### Glucosinolate hydrolysis product composition

**Figure 4:**
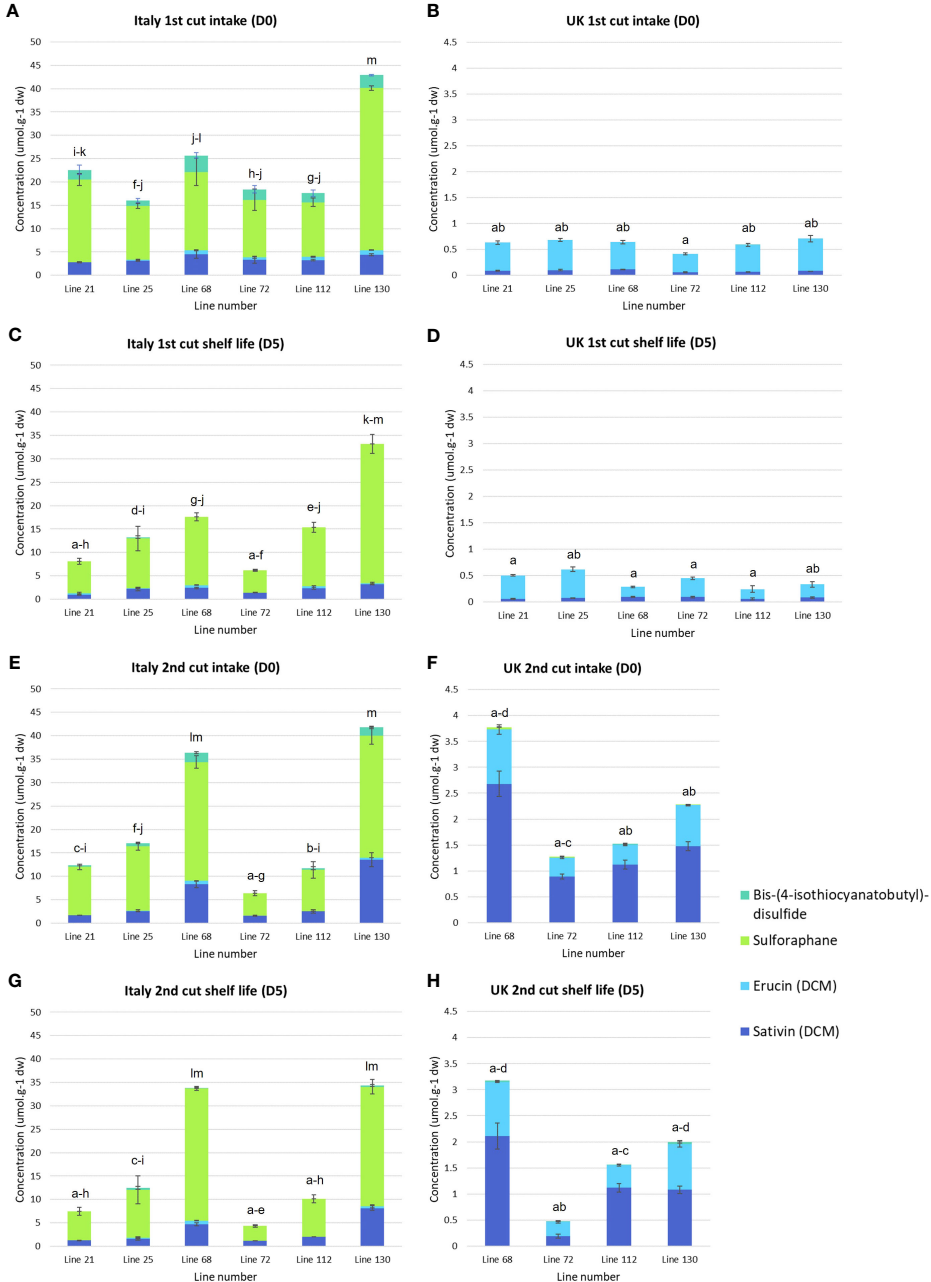
Semi-volatile glucosinolate hydrolysis product concentrations of six *Eruca vesicaria* subsp. *sativa* extreme lines grown in Italy and the UK. Error bars represent standard error of the mean for each compound. Letters above bars indicate significant differences observed for total glucosinolate hydrolysis product concentrations between each sample and environment. Note the difference in y-axis scale between Italy and UK samples **(B, D, F, H)**. **(A)** Italy, first cut intake (D0); **(B)** UK, first cut intake (D0); **(C)** Italy, first cut shelf life (D5); **(D)** UK, 1st cut shelf life (D5); **(E)** Italy, second cut intake (D0); **(F)** UK, second cut intake (D0); **(G)** Italy, second cut shelf life (D0); **(H)** UK, second cut shelf life (D5).

Significant differences were observed in the concentrations of semi-volatile GHPs (**Supplementary File S3**, **Figure 4**) between Italy and UK samples for all *Eruca* lines. Very low concentrations were observed for all UK samples, with concentrations in Italy-grown plants over 400-fold greater in one instance (line 130, 1^st^ cut D0). This reflects the trend observed for GSLs (**Figure 3**). The dominant compounds in extracts were SF and SAT in Italy-grown leaves, and SF and ERU in UK leaves. Lines 68 and 130 produced the highest concentrations at each time point of the Italy trial (36.4 μmol g^-1^ DW, Italy 2^nd^ cut D0, and 42.9 μmol g^-1^ DW, Italy 1^st^ cut D0, respectively; **Figure 4**). The overall difference in GSL and GHP concentrations observed between the two locations is reflected in sulfur content, with Italy samples having significantly higher amounts than the UK (**Figure 1**).

### Plant gene expression and co-expression

**Figure 5:**
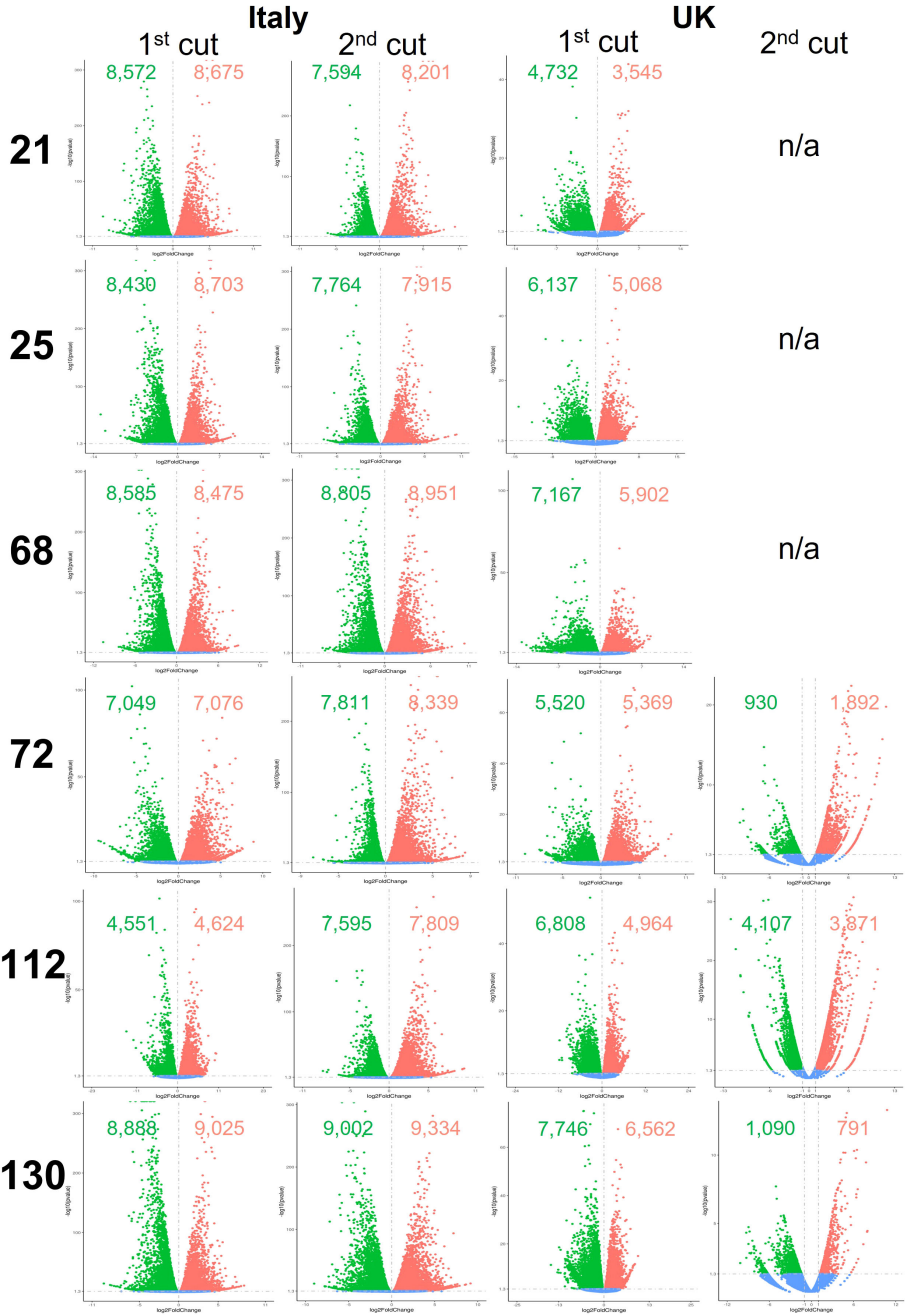
Differentially expressed genes between shelf-life time points (D0 and D5) of six *Eruca vesicaria* subsp. *sativa* extreme lines grown in Italy and the UK. Red dots represent significantly up-regulated genes. Green dots represent significantly down-regulated genes. Blue dots represent non-significant differentially expressed genes. Second cut shelf-life samples for lines 21, 25 and 68 grown in the UK were omitted from the analysis because RNA of sufficient quality could not be obtained for these samples. n/a, not applicable.

**Figure 6:**
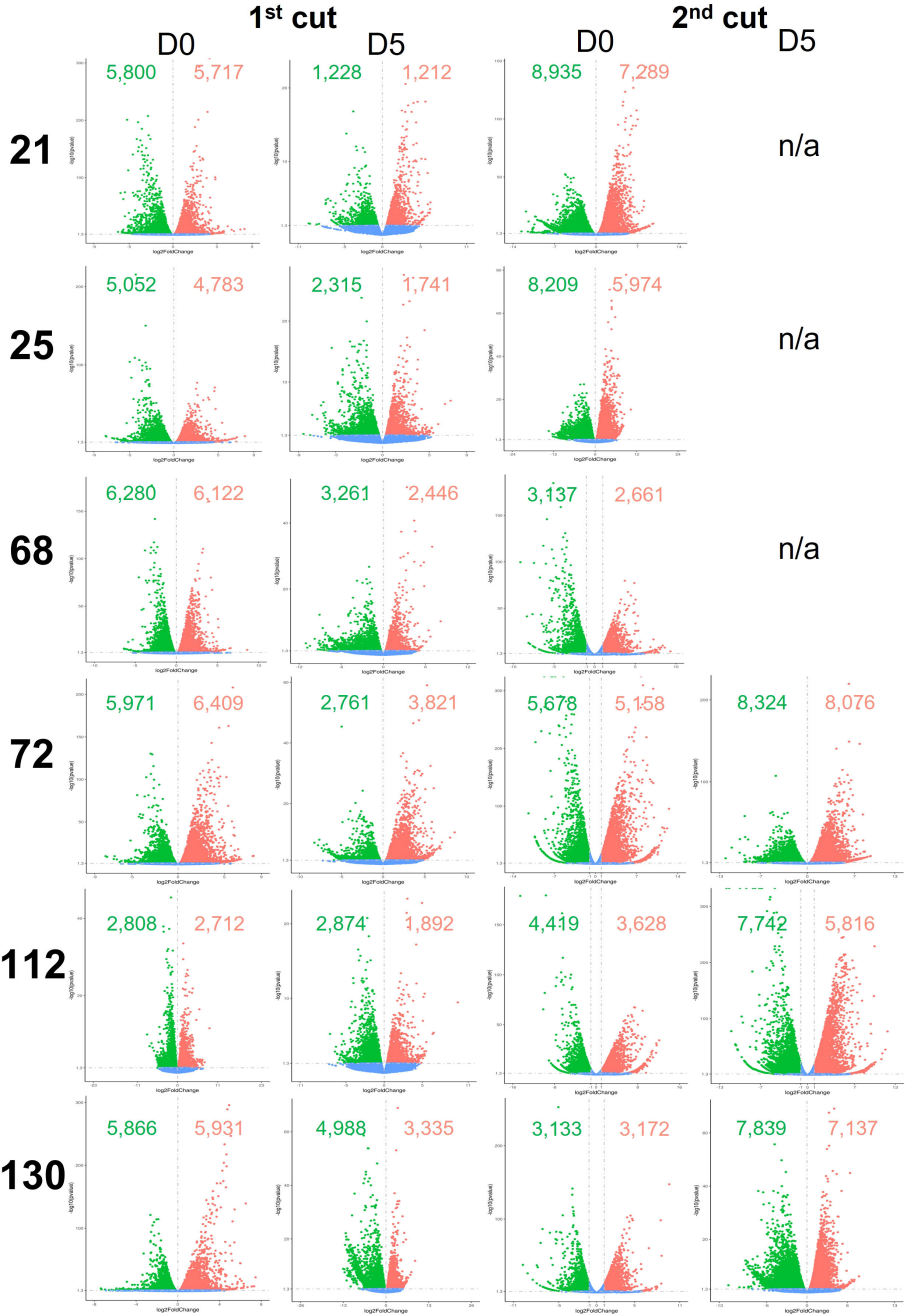
Differentially expressed genes between growing location (Italy and UK) of six *Eruca vesicaria* subsp. *sativa* extreme lines grown in Italy and the UK. Red dots represent significantly up-regulated genes. Green dots represent significantly down-regulated genes. Blue dots represent non-significant differentially expressed genes. Second cut shelf-life samples for lines 21, 25 and 68 grown in the UK were omitted from the analysis because RNA of sufficient quality could not be obtained for these samples. n/a, not applicable.

Global differential gene expression in *Eruca* samples between plants grown in the two countries are presented in **Figure 5**, and between D0 and D5 samples in **Figure 6**. Data presented in **Figure 5** are of UK samples relative to Italy. Data presented in **Figure 6** are of D5 samples relative to D0. 2^nd^ cut D5 samples for lines 21, 25, and 68 were omitted due to poor RNA integrity in at least one replicate of UK samples.

Numbers of differentially expressed genes (DEGs) between plants grown in the two countries and time points are highly genotype dependent. When looking at 1^st^ cut D0, line 112 showed few DEGs (2,712 up, 2,808 down) compared with line 68 (6,122 up, 6,280 down; **Figure 6**). Different trends were observed in the 2^nd^ cut (D0), where line 68 had a lower number of DEGs (2,661 up, 3,137 down) compared with lines such as 21 (7,289 up, 8,935 down). Expression was more uniform in D5 1^st^ cut than for D5 2^nd^ cut. Lines 72, 112, and 130 showed a 2.1-fold, 3.1-fold, and 2.1-fold change in up-regulated genes (respectively), and a 3.0-fold, 2.7-fold, and 1.6-fold change in the numbers of down-regulated genes (respectively).

DEGs between D0 and D5 samples within each respective country (**Figure 5**) were high, indicating changes in cellular metabolism postharvest. Two notable exceptions to this were 2^nd^ cuts of lines 72 and 130 in the UK trial. Gene expression was consistent in these samples, with 72 having 1,892 up-regulated genes and 930 down-regulated, and 130 having 791 up-regulated genes and 1,090 down-regulated. This represents a 2.8-fold and 5.9-fold difference (respectively) for 72, and an 8.3-fold and 7.1-fold difference (respectively) for 130 when compared with the UK 1^st^ cut leaves.

**Figure 7:**
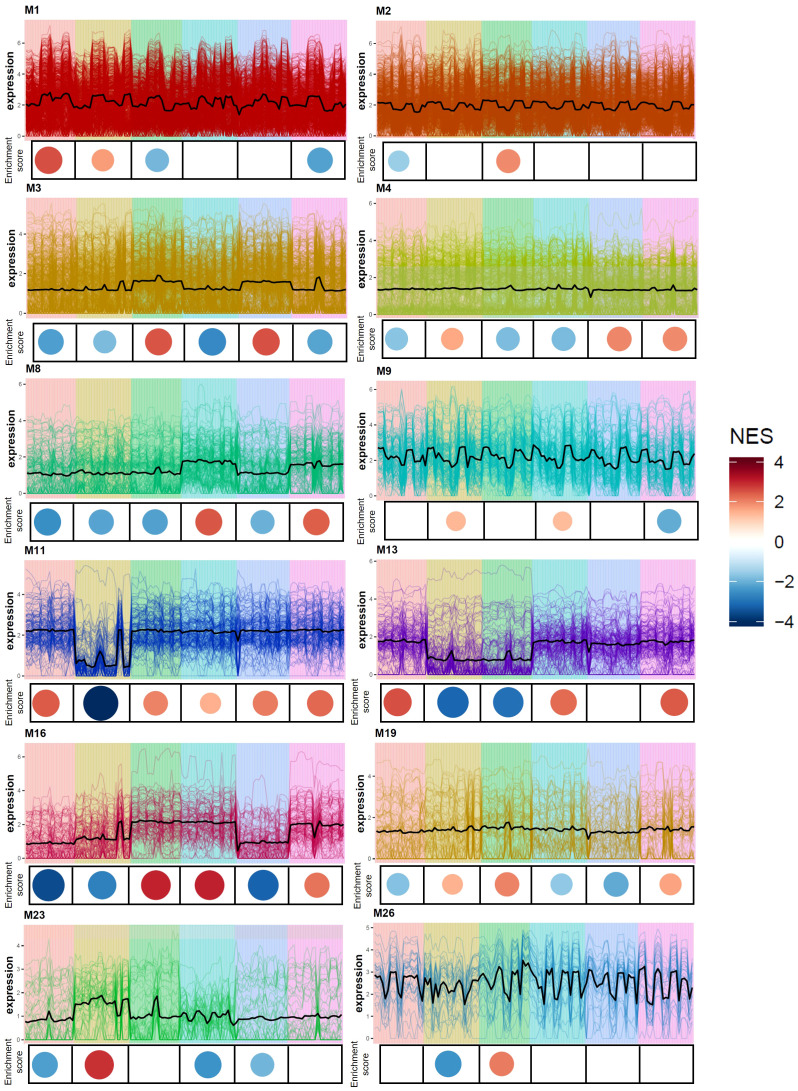
Co-expression gene expression analysis of six *Eruca vesicaria* subsp. *sativa* extreme lines. Line charts illustrate expression (fpkm) of genes within each respective module. Coloured circles beneath each line chart signify the normalized enrichment score (NES) of the six *Eruca* lines. Red signifies enriched up-regulated gene expression, and blue indicates down-regulated gene expression. Values are relative across all co-expression modules.

**Figure 8:**
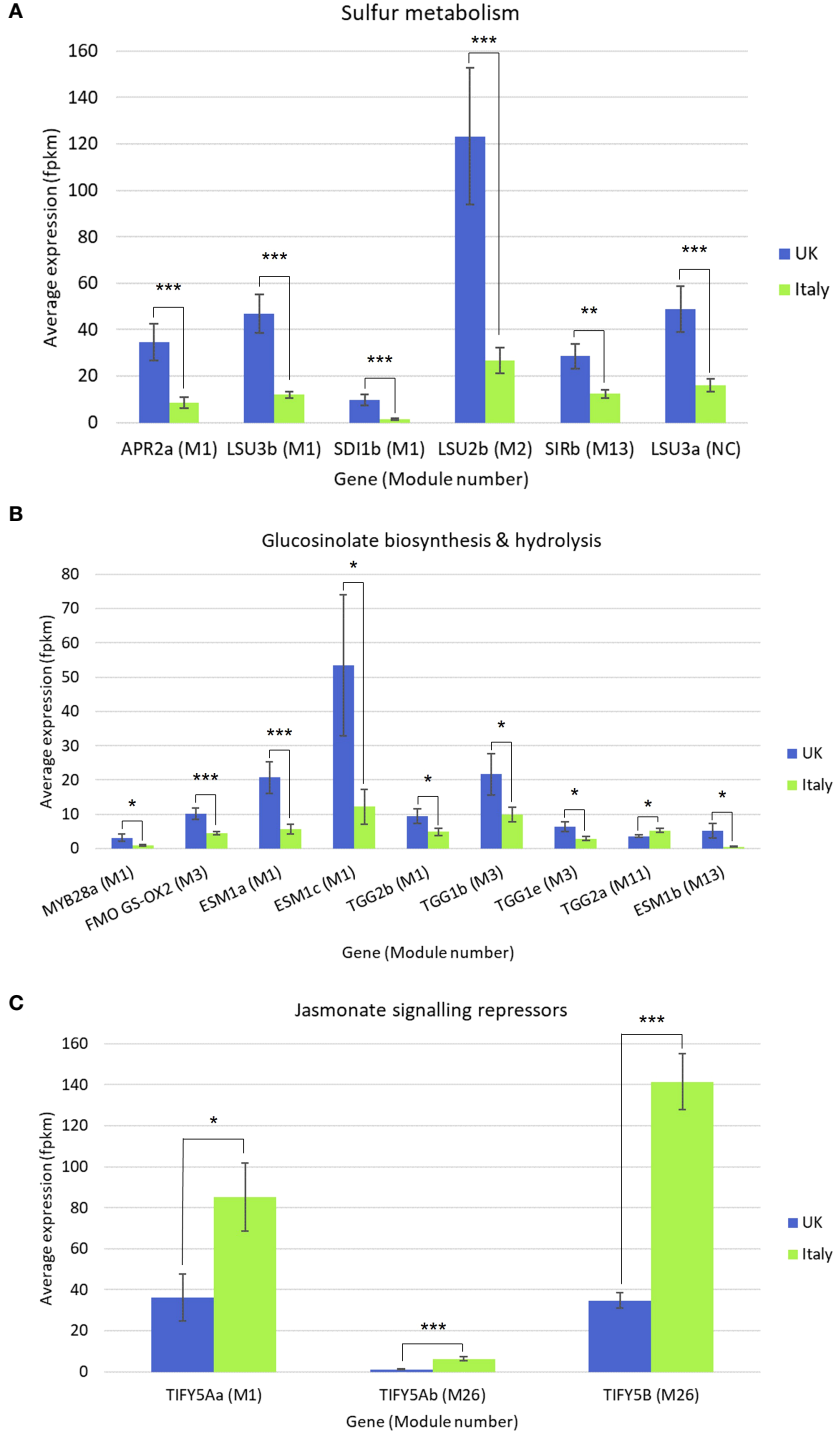
Differentially expressed genes (DEGs) in *Eruca* plants grown in Italy and the UK. Average expression values (fpkm) are presented for **(A)** sulfur metabolism, **(B)** glucosinolate biosynthesis and hydrolysis, and **(C)** jasmonate signaling repressor genes. Module number refers to co-expression analysis results which can be found in **Supplementary File S6**. Error bars represent standard error of the mean. Asterisks denote level of significant difference: *p<0.05; **p<0.01; ***p<0.001; See insets for colour coding.

Gene co-expression analysis produced 28 modules of varying size (**Supplementary File S6**) across the six *Eruca* lines. 12 modules were found to contain genes associated with sulfur metabolism, GSL biosynthesis, GSL hydrolysis, glutathione metabolism, and defense response. Relative expression and enrichment scores for each line are presented in **Figure 7**. Several genes contained within co-expression modules were found to be significantly differentially expressed between Italy and UK across all leaf samples. **Figure 8** shows gene expression for sulfur metabolism, GSL biosynthesis and hydrolysis, and jasmonate (JA) signaling repressors. In nearly all cases expression of these genes was significantly higher in UK-grown plants. The largest co-expression module (M1) contained *APR2a*, *LSU3b*, *SDI1b*, *MYB28a*, *ESM1a*, *ESM1c*, and *TGG2b*. The only exception was *TIFY5Aa*, which was expressed significantly more in Italy-grown plants. Two other JA repressors, *TIFY5Ab* and *TIFY5B* (M26), also displayed this trend. *TIFY5B* had 4.6-fold greater expression in Italy leaf samples than UK, for example. Correlation analysis found distinct clusters of associated expression between genes contained in the co-expression modules (**Supplementary Files S6**) which could be used to develop panels of expression markers for associated quality traits.

### Principal component analysis

**Figure 9:**
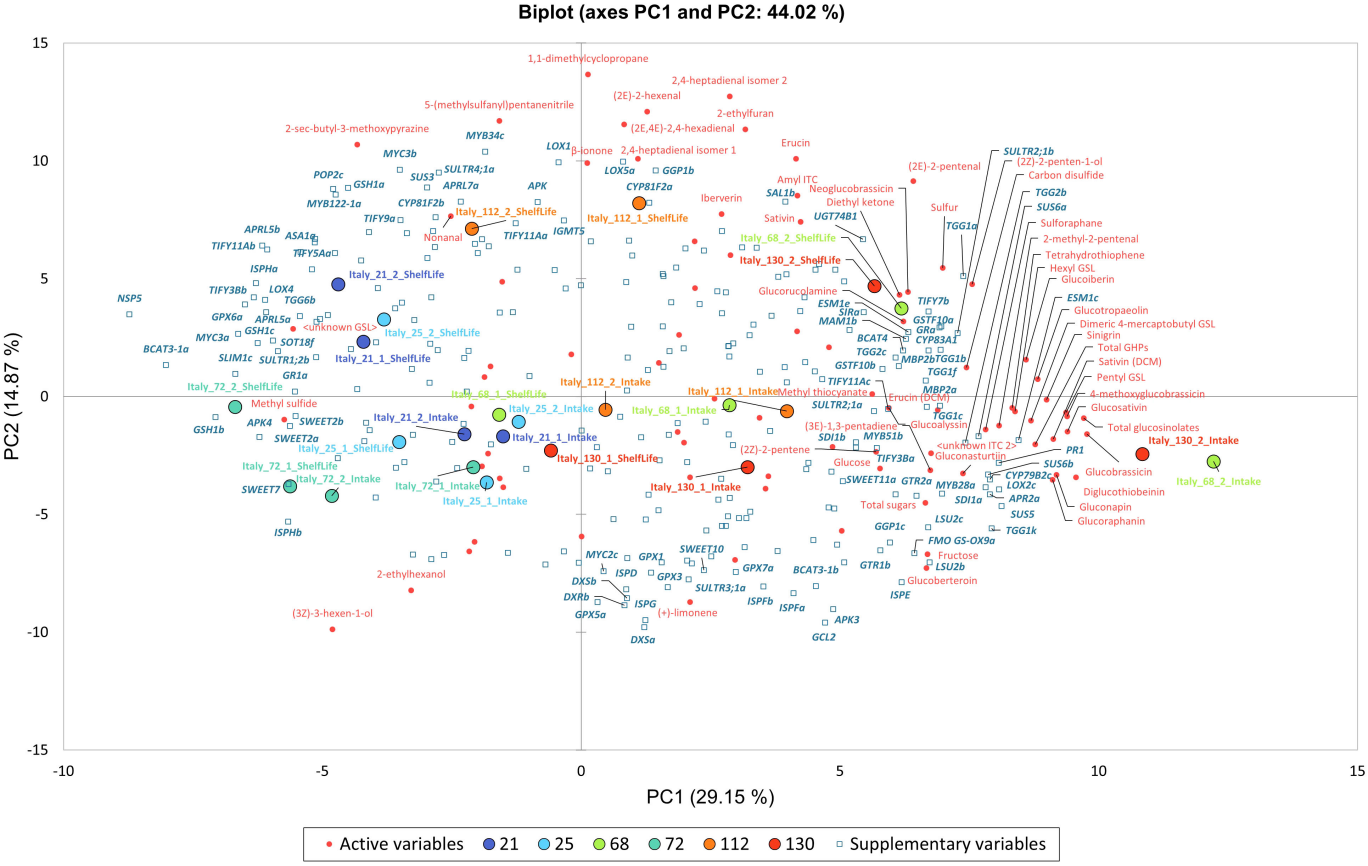
Principal Component Analysis (PCA) biplots for Italy-grown *Eruca vesicaria* subsp. *sativa* extreme line sample phytochemical composition values. PC1 and PC2 were selected for presentation and explain 44% of the observed variation. The biplot presents phytochemical data (red text) association with gene expression (fpkm; bold blue italic text). See inset for colour coding of cultivar samples.

**Figure 9** presents a PCA of Italy-grown plants’ phytochemical composition and expression levels (fpkm) for selected genes of interest. Italy data were analysed because of the range of observed responses from cultivars, and the completeness of RNAseq data. The PCA explains 44% of the observed variability between samples (PC1, 29.2%; PC2, 14.9%). PC1 and PC2 eigenvalues were >10 and selected for presentation as they explained the greatest percentage variance. PC1 separates primarily for GSL concentrations (total, GBC, DGTB, GSV, 4MGB, and PEN) and PC2 for VOC abundances (1,1-dimethylcyclopropane, 2,4-heptadienal, (2*E*)-2-hexenal, 5-(methylsulfanyl)pentanenitrile, (2*E*,4*E*)-2,4-hexadienal, and 2-ethylfuran).

Samples separate along PC1 for GSL concentrations with the ‘high’ accumulating lines (68 and 130) present in the right two quadrants, and ‘low’ accumulating lines (21, 25, and 72) on in the left two quadrants. Line 112 samples are loosely clustered in the center of the biplot. To the extreme right of the biplot are 68 and 130 2^nd^ cut D0 samples, with their D5 counterparts located above and to the left in the upper right quadrant. These samples are associated with GSL abundances, hydrolysis products (SAT), and sulfur-containing VOCs (tetrahydrothiophene and carbon disulfide). Abundances of these compounds are associated with expression of genes relating to GSL biosynthesis (*CYP79B2c*), GSL hydrolysis (*ESM1c, TGG1k*), sugar metabolism (*SUS5, SUS6a, SUS6b*), VOC synthesis (*LOX2c*), plant defense (*PR1*), and sulfur metabolism (*APR2a, SDI1a*). Conversely, on the negative axis of PC1 (associated with lower GSL concentrations) lines 21, 25 and 72 are associated with low expression of the aforementioned genes, and higher expression of others involved in GSL biosynthesis (*BCAT3-1a*), GSL hydrolysis (*NSP5*), glutathione metabolism (*GSH1b, GSH1c, GPX6a*), sulfur limitation (*SLIM1c*), sulfur metabolism (*APK4*), jasmonic acid-related stress response (*MYC3a, TIFY3Bb*), and VOC synthesis (*ISPHa*).

Correlation analysis of gene expression highlighted in co-expression analysis revealed strong and significant (*p* = <0.001) associations with phytochemical metabolites. A summary table of *r* values is presented in **Table 3** (see **Supplementary File S5** for the full correlation data set). Expression of *APR2a* and two copies of *SDI1* (*SDI1a* and *SDI1b*) had strong positive correlations with GSLs, such as GNP (*r* = >0.661), GBC (*r* = >0.646), 4MGB (*r* = >0.667), and GNT (*r* = >0.63), as well as abundance of carbon disulfide (*r* = >0.754). The expression of *MYB28a* and *CYP79B2c* was also significantly correlated with the abundance of several GSLs (GRA, *r* = >0.716; GNP, *r* = >0.655; DGTB, *r* = >0.741; GSV, *r* = >0.668). Expression of the myrosinases *TGG1k* and *TGG2b*, and modifier protein encoding *ESM1c* were significantly correlated with concentrations of SAT (*r* = >0.742). These relationships are suggestive of a suite of genes in high GSL/GHP lines which give rise to their phenotype, rather than a single gene controlling the process in isolation. These positive relationships contrast with *APK4, BCAT3-1a*, *NSP5* and *SLIM1c* where strong negative correlations with sulfur containing metabolites were observed. Of note are negative correlations with glutathione-related genes, *GPX6a, GSH1b* and *GSH1c*, as it has been previously hypothesised that glutathione metabolism and GSL biosynthesis are competing pathways for sulfur (Vanegas Torres et al., 2022).

**Table 3:** Pearson’s Correlation coefficients (*r*) of *Eruca vesicaria* subsp. *sativa* gene expression (fpkm) with phytochemical metabolites from six extreme lines cultivated in Italy.

Compound	Sulfur metabolism					
	*APK4*	*APR2a*	*SDI1a*	*SDI1b*	*SLIM1c*	
Glucosinolates						
GIB	ns	0.634	ns	ns	-0.803	
SIN	ns	0.769	0.734	ns	-0.629	
GRA	ns	0.743	0.731	ns	ns	
GAL	-0.635	ns	ns	ns	ns	
GNP	ns	0.868	0.843	0.661	ns	
DGTB	ns	0.843	0.798	ns	-0.643	
GBT	ns	0.700	ns	ns	ns	
GSV	ns	ns	0.808	ns	ns	
DMB	ns	ns	ns	ns	-0.731	
GTP	ns	ns	ns	ns	-0.683	
GBC	ns	0.864	0.839	0.646	ns	
4MGB	ns	0.851	0.824	0.667	ns	
GNT	ns	0.903	0.933	0.630	ns	
HEX	-0.660	0.644	0.724	ns	ns	
Total GSLs	ns	0.633	ns	ns	-0.713	
GSL hydrolysis products						
SAT	ns	0.656	0.707	ns	ns	
SF	-0.652	ns	ns	ns	ns	
Total GHPs	-0.661	ns	ns	ns	ns	
VOCs						
(2*Z*)-2-pentene	ns	0.657	ns	0.677	ns	
Methyl sulfide	ns	ns	ns	ns	0.642	
Carbon disulfide	ns	0.754	0.772	0.768	ns	
2-methyl-2-pentenal	ns	0.657	0.687	ns	ns	
	Glucosinolate biosynthesis			Glutathione metabolism		
	*BCAT3-1a*	*CYP79B2c*	*MYB28a*	*GPX6a*	*GSH1b*	*GSH1c*
Glucosinolates						
GIB	-0.667	ns	ns	ns	-0.666	ns
PEN	-0.705	0.683	ns	-0.645	-0.634	ns
SIN	-0.765	ns	0.650	ns	-0.636	ns
GRA	-0.721	0.759	0.716	ns	ns	ns
GNP	ns	0.655	0.802	ns	ns	ns
DGTB	-0.692	0.741	0.790	-0.748	ns	ns
GBT	ns	ns	ns	-0.629	ns	ns
GSV	-0.646	0.694	0.668	ns	-0.665	ns
DMB	-0.737	ns	ns	ns	ns	-0.650
GTP	-0.759	0.641	ns	-0.650	-0.670	-0.660
GBC	-0.694	ns	0.735	ns	ns	ns
4MGB	-0.655	ns	0.707	ns	ns	ns
GNT	ns	ns	0.745	ns	ns	ns
HEX	-0.688	0.707	ns	ns	ns	ns
Total GSLs	-0.772	0.674	ns	ns	-0.665	-0.634
GSL hydrolysis products						
SAT	-0.648	0.711	ns	ns	ns	ns
SF	ns	0.725	ns	ns	-0.638	ns
Bis-(4-isothiocyanatobutyl)-disulfide	ns	0.665	ns	-0.646	ns	ns
Total GHPs	-0.664	0.797	ns	ns	-0.668	ns
VOCs						
Methyl sulfide	ns	ns	ns	ns	0.661	ns
2-methyl-2-pentenal	ns	0.630	0.667	ns	ns	ns
(+)-limonene	ns	ns	ns	-0.675	ns	ns
<unknown ITC 2>	ns	0.658	ns	ns	ns	ns
	Glucosinolate hydrolysis				VOC synthesis	
	*ESM1c*	*NSP5*	*TGG1k*	*TGG2b*	*ISPHa*	*LOX2c*
Glucosinolates						
GIB	ns	-0.782	ns	ns	ns	ns
PEN	0.679	-0.832	0.751	0.828	-0.630	0.703
PRO	ns	ns	ns	ns	ns	0.640
SIN	0.780	-0.748	0.704	ns	ns	0.701
GRA	0.802	-0.709	0.671	0.635	ns	0.770
GRM	0.675	ns	ns	ns	ns	ns
GNP	0.871	-0.747	0.822	ns	ns	0.837
DGTB	0.791	-0.864	0.865	0.670	-0.654	0.800
GBT	ns	-0.716	0.792	ns	-0.701	ns
GSV	0.867	-0.730	0.798	0.771	ns	0.878
DMB	ns	-0.826	0.631	0.632	ns	ns
GTP	ns	-0.819	ns	0.637	ns	ns
GBC	0.857	-0.807	0.835	ns	-0.640	0.815
4MGB	0.825	-0.740	0.799	ns	ns	0.786
GNT	0.920	ns	0.813	ns	ns	0.933
HEX	0.762	-0.688	0.683	0.695	ns	0.733
Total GSLs	0.748	-0.861	0.730	0.720	ns	0.677
GSL hydrolysis products						
SAT	0.742	-0.739	0.797	0.857	-0.640	0.852
SF	ns	ns	ns	0.739	ns	ns
Total GHPs	ns	-0.708	ns	0.820	ns	ns
VOCs						
(3*E*)-1,3-pentadiene	0.644	ns	ns	ns	ns	0.701
Carbon disulfide	0.664	ns	ns	ns	ns	ns
2-methyl-2-pentenal	0.838	ns	0.630	ns	ns	0.698
(4*Z*)-4-heptenal	ns	ns	ns	0.673	ns	ns
Tetrahydrothiophene	ns	-0.730	0.652	ns	ns	0.630
Sugars						
Fructose	ns	-0.652	0.736	ns	ns	0.647
Total sugars	ns	ns	0.668	ns	-0.659	ns
	Jasmonic acid signaling/stress response					
	*MYC3a*	*PR1*	*TIFY3Bb*	*TIFY5Aa*	*TIFY5Ab*	*TIFY5B*
Glucosinolates						
GIB	-0.710	ns	-0.726	ns	ns	ns
PEN	ns	0.732	ns	ns	ns	ns
PRO	ns	0.722	ns	ns	ns	ns
SIN	ns	0.740	ns	ns	ns	ns
GRA	ns	0.784	ns	ns	ns	ns
GNP	ns	0.828	ns	ns	ns	ns
DGTB	ns	0.763	-0.709	ns	ns	ns
GBT	-0.718	0.650	-0.769	ns	ns	ns
4HGB	ns	ns	ns	ns	ns	-0.640
GSV	ns	0.866	ns	ns	ns	ns
GBC	ns	0.811	ns	ns	ns	ns
4MGB	ns	0.806	ns	ns	ns	ns
GNT	ns	0.862	ns	ns	ns	ns
HEX	ns	0.723	ns	ns	ns	ns
<unknown GSL>	ns	ns	ns	0.674	ns	ns
Total GSLs	ns	0.681	ns	ns	ns	ns
GSL hydrolysis products						
SAT	ns	0.873	ns	ns	ns	ns
Total GHPs	ns	0.683	ns	ns	ns	ns
VOCs						
Methyl sulfide	ns	ns	ns	0.718	0.706	ns
3-methylfuran	ns	ns	ns	0.675	ns	ns
(3*E*)-1,3-pentadiene	ns	0.666	ns	ns	ns	ns
2-methyl-2-pentenal	ns	0.690	ns	ns	ns	ns
(+)-limonene	ns	ns	-0.724	ns	ns	ns
Sugars						
Fructose	-0.654	ns	-0.678	ns	ns	ns
Total sugars	-0.680	ns	-0.644	ns	ns	ns
	Sugar metabolism					
	*SUS5*	*SUS6a*	*SUS6b*			
Glucosinolates						
GIB	0.688	ns	ns			
PEN	0.763	0.682	0.694			
GRA	0.662	0.694	0.707			
GNP	0.822	0.635	0.772			
DGTB	0.901	0.740	0.824			
GBT	0.736	ns	0.712			
GSV	0.738	0.674	0.705			
DMB	0.716	ns	ns			
GTP	0.669	0.689	ns			
GBC	0.761	0.631	0.731			
4MGB	0.697	ns	0.682			
GNT	0.672	ns	0.642			
HEX	0.632	ns	0.639			
Total GSLs	0.779	0.685	0.675			
GSL hydrolysis products						
SAT	0.747	0.692	0.754			
SF	ns	0.661	ns			
Bis-(4-isothiocyanatobutyl)-disulfide	ns	ns	0.715			
Total GHPs	ns	0.737	0.669			
VOCs						
2-methyl-2-pentenal	0.686	0.709	0.687			
Tetrahydrothiophene	0.679	ns	ns			
<unknown ITC 2>	0.665	0.672	ns			
Sugars						
Fructose	0.741	ns	ns			
Total sugars	0.669	ns	ns			

Genes were selected based on the results of co-expression analysis and Principal Component Analysis. All correlations are significant to p <0.001. Full results are presented in **Supplementary Data File S5** and **Supplementary File S6**. GSL, glucosinolate; GHP, glucosinolate hydrolysis product; VOC, volatile organic compound; ITC, isothiocyanate; GIB, glucoiberin; PEN, pentyl GSL; PRO, progoitrin; SIN, sinigrin; GRA, glucoraphanin; GRM, glucorucolamine; GNP, gluconapin; DGTB, diglucothiobeinin; GBT, glucoberteroin; 4HGB, 4-hydroxyglucobrassicin; GSV, glucosativin; DMB, dimeric 4-mercaptobutyl GSL; GTP, glucotropaeolin; GBC, glucobrassicin; 4MGB, 4-methoxyglucobrassicin; GNT, gluconasturtiin; HEX, hexyl GSL; SAT, sativin; SF, sulforaphane. ns, not significant.

*APR2a, SDI1a, BCAT3-1a*, and *NSP5* have been highlighted as having associations with GSL concentrations in the parental lines of the *Eruca* lines tested here (Bell et al., 2020a). This is evidence of the heritable nature of GSL compositions between environments (parental lines were grown in controlled environment chambers) and that these specific genes could act as useful expression markers for GSL concentrations.

## Discussion

### Growing environment determines *Eruca* sugar concentrations and impacts shelf-life quality

Consistent with a previous study (Bell et al., 2022), cultivation environment has a significant impact on the accumulation of sugars. This has clear implications for the taste of leaves, and in this study, it was observed that the accumulation of sugars is inversely related to concentrations of sulfur and GSL concentrations in Italy and UK-grown plants. We hypothesised that increased sugar concentrations in UK-grown plants may be reflective of cultivation practice (open field compared with polytunnel in Italy) and that respiration rates and levels of stress are increased because of the cooler, more humid climate (Bell et al., 2020b).

Another possible contributing factor to the disparities in sugar concentrations could be the intensity and duration of daylight between the two growing locations. With the UK being at a higher latitude, the rocket crop received an average of 15.9 daylight hours compared with an average 12 daylight hours in Italy at the times of each respective trials. Experiments in controlled environment have shown that sugars increase in kale when grown under long photoperiods (Steindal et al., 2015). In combination with the impacts of temperature and humidity, the role light plays in sugar accumulations of rocket may be significant, and worthy of more detailed investigation in future.

Sugar accumulation is also known to be associated with stress and the initiation of senescence (Sami et al., 2016), and in *Eruca* the shortening of shelf-life due to these processes results in lower nutritional quality of leaves. An explanation may be the link between sugar accumulation and acclimatization to adverse environmental conditions through oxidative stress response. Glucose is a known inhibitor of lipid peroxidation (Sami et al., 2016) and high accumulations in leaves may therefore be indicative of the stressful environment and metabolic efforts to slow the degradation of tissues. Combined with lower S-availability, this results in low GSL concentrations in UK crops and a reduction in nutritional quality.

### Volatile organic chemical profiles are indicative of increased postharvest stress in UK-grown *Eruca*

Changes in VOCs during shelf-life can be used as markers of quality and to elucidate biological processes. Rocket species have been researched extensively in this area and it is known that preharvest factors significantly influence the types of compounds produced. Research conducted on *Diplotaxis tenuifolia* leaves has shown that increases in carbon disulfide and 3-methylfuran are associated with prolonged shelf-life storage and cellular deterioration. It has been suggested that 3-methylfuran has a fungal origin, and may therefore be indicative of leaf spoilage (Luca et al., 2016). In this study the relative abundance of these compounds was found to be significantly impacted by cut number and shelf-life duration between countries, being highest in UK 1^st^ cut D0 samples. Such abundances in early shelf life indicates a high level of cellular degradation, even in freshly harvested material, signifying its low quality. Replicates of lines 21 and 25 had to be omitted from analyses due to the low quality of RNA, for example. This supports our hypothesis that UK-grown plants were suffering increased oxidative stress during growth, thereby accelerating senescence and degradation processes. As well as using specific VOCs as potential markers of *Eruca* quality, the ratios between VOC components may be indicative (**Figure 2**). D0 and D5 UK samples showed low abundances of alcohols, and increased amounts of alkanes and aldehydes compared to Italy samples at the same respective time points. This is suggestive of lipid breakdown and high amounts of tissue wounding (Brecht, 1995) which would severely reduce shelf-life viability.

### Gene co-expression and sulfur content is associated with greater glucosinolate concentrations and hydrolysis product formation

The low GSL concentration in UK-grown *Eruca* is associated with lower sulfur found within leaves. The metabolic link between the two is well established, with GSLs acting as a form of sulfur storage molecule as well as a means of chemical defense (Morikawa-Ichinose et al., 2019). Our data show that several sulfur assimilation genes (**Figure 8**) have significantly higher expression in UK plants compared to Italy, including *SDI1b* (*SULFUR DEFICIENCY INDUCED 1*). This indicates that UK-grown plants were in a state of sulfur deficiency, and thus unable to synthesize greater quantities of GSLs. Under these conditions there would be high rates of GSL turnover to conserve sulfur (Maruyama-Nakashita, 2017). The high expression of *TGG1* and *TGG2* myrosinases is indicative of this (Ristova and Kopriva, 2022; **Figure 8**), although not itself a measure of enzyme abundance or activity. If the hypothesis of high oxidative stress in UK-grown *Eruca* is correct, the limitation of sulfur may further impair the quality of leaves at harvest and throughout shelf-life, as molecular sulfur itself is known to be a key component in regulating physiological responses to stress (Chan et al., 2019). Another contributing factor to the differences in GSL concentrations between the two locations may also be light intensity and duration. Light components (such as UV and visible wavelengths) are well known to affect the concentrations of some GSLs (Zhou et al., 2023), and recent reports suggest that this may be of particular importance in the synthesis of DMB and DGTB (Dernovics et al., 2023). The relatively higher-light environment in Italy may have been a contributing factor to the abundance of GSLs alongside the greater availability of sulfur.

Expression of some genes previously shown to be up-regulated under S-deprivation did not follow this pattern in our experiment. For example, *PR1* is known to play a role in secondary metabolism through regulation of the salicylic acid pathway and distribution of sulfur and it was up-regulated under S-limited conditions in a previous study using *Arabidopsis* (Criollo-Arteaga et al., 2021). In our study *PR1* was highly expressed in Italy grown plants and associated with high GSL concentrations (**Figure 9**; **Table 3**). Sulfur was not limiting and was significantly higher in leaves than all UK-grown samples (**Figure 1**). *PR1* may therefore function differently in *Eruca* and in fact be up regulated when sulfur is not limited.

Co-expression analysis and PCA has highlighted several genes for further study (e.g., *APR2, SDI1, BCAT3, ESM1* and *NSP5*, and which could be utilized as expression markers in future research and breeding efforts. As demonstrated here, expression of specific gene copies related to sulfur assimilation, GSL biosynthesis, and GHP formation must be considered together, as no single gene appears to be responsible for increased concentrations. Indeed, it has been demonstrated that high/low GSL abundances are heritable traits in *Eruca*, but that cultivation environment significantly impacts the ability of plants to synthesize health-related secondary metabolites. Panels of expression markers based on these data could be developed for screening *Eruca* cultivars across a broad range of locations without the need for expensive and time-consuming chemical analyses. This would allow growers to better select cultivars that are well suited to their climate and that will perform best in their production environment.

## Study limitations and future research

This study was limited to only a single trial in each of the two countries. It was unfortunately not possible to conduct follow-up experiments and establish inter-year variability of rocket gene expression and metabolite profiles due to the Covid-19 pandemic.

Future research should address the link between cultivation environment and generation of GHPs related to human health and consumer acceptability. Our research has identified potential gene candidates for further study, and manipulation of expression through use of RNA interference or CRISPR-Cas9 genome editing may reveal the functionality and contribution of individual genes to the regulation or promotion of GHP formation. Many of the genes responsible for synthesis of GSL compounds (e.g., GSV, DMB, DGTB, GRM) are unidentified, and there is significant scope for expanding research and knowledge of GSL biosynthesis through utilization of the *Eruca* genome (Bell et al., 2020a).

## Conclusion

Unpicking the effects of different environmental parameters (such as temperature) on transcriptomic changes and phytochemical abundances is challenging under field conditions. This study has attempted to understand the wholistic impacts of cultivation location on phytochemical and nutritional quality and is reflective of ‘real world’ production and what consumers might realistically experience when eating *Eruca* leaves. We have demonstrated that the environment has significant effects on the amounts of phytochemicals people may receive in their diets depending on rocket genotype and where it has been cultivated. This is a perennial issue for processors in terms of related traits, such as flavour and postharvest longevity. As demonstrated here, it makes it almost impossible to guarantee consistency of *Eruca* phytochemicals when sourced from multiple locations. As with the wine industry, cultivars should be produced in specific locales according to the distinctive traits generated through the interaction of specific genotypes with the environment. This will allow growers and processors to generate high value and distinctive products that can be produced with much better consistency.

## Data availability statement

The datasets presented in this study can be found in online repositories. The names of the repository/repositories and accession number(s) can be found in the article/**Supplementary Material**.

## Author contributions

Conceptualization of the research was by LB, LM, and CW. Methodology was designed by LB, LM, RT, and CW. Formal analysis was conducted by LB, MC, MP, and JJ. Field trials were organized by LB and MC. Analysis of VOCs by GC-MS was performed by JJ. GSL analysis by LC-MS and GHP analysis by GC-MS was performed by LB. Sulfur extraction and analysis by ICP-OES was performed by MC. Sugar analysis by HPIC-MS was performed by MC and MP. RNA extractions were performed by MC. Transcriptome co-expression analysis was performed by LB. Phytochemical statistical analyses were performed by LB. Resources, such as *Eruca* seed, was provided by RT. Data curation was performed by LB. Writing of the original manuscript draft was done by LB. Additional writing, review and editing was provided by MC, MP, LM, and CW. Visualization of data was performed by LB. Supervision of the research was provided by LM and CW. Project administration was organized by LB, MC, and CW. Funding acquisition was performed by LB, LM, and CW. All authors contributed to the article and approved the submitted version.

## Supplementary material

Supplementary File S1 Sampling diagram.

Supplementary File S2 List of Volatile Organic Compounds (VOCs) identified in six *Eruca vesicaria* subsp. *sativa* extreme lines grown in Italy and the UK.

Supplementary File S3 Summary table of Analysis of Variance (ANOVA) with *post-hoc* Tukey’s Honest Significant Difference (HSD) results for *Eruca vesicaria* subsp. *sativa* phytochemical compositions.

Supplementary File S4 Sugar concentrations of six *Eruca vesicaria* subsp. *sativa* extreme lines grown in Italy and the UK. Error bars represent standard error of the mean for each compound. Letters above bars indicate significant differences observed for total sugar concentrations between each sample and environment.

Supplementary File S5 Summary of genes identified within each co-expression module of six *Eruca vesicaria* subsp. *sativa* extreme lines, with KEGG annotations.

Supplementary File S6 Pearson’s correlation analysis (*n*-1) results for six *Eruca vesicaria* subsp. *sativa* extreme line phytochemical compositions and selected gene expression data (related to sulfur metabolism, glucosinolate biosynthesis, glucosinolate hydrolysis, redox metabolism, VOC synthesis, stress response, and sugar metabolism). Values in bold are significant correlations *p* = <0.001. Red cells signify negative correlations, green cells signify positive correlations.
