# Supplementary figures and images for: Genotypes of Eruca vesicaria subsp. sativa grown in contrasting field environments differ on transcriptomic and metabolomic levels, significantly impacting nutritional quality
Source: Front Plant Sci. 2023 Nov 2;14:1218984. doi: 10.3389/fpls.2023.1218984 (PMC10652768; doi:10.3389/fpls.2023.1218984)

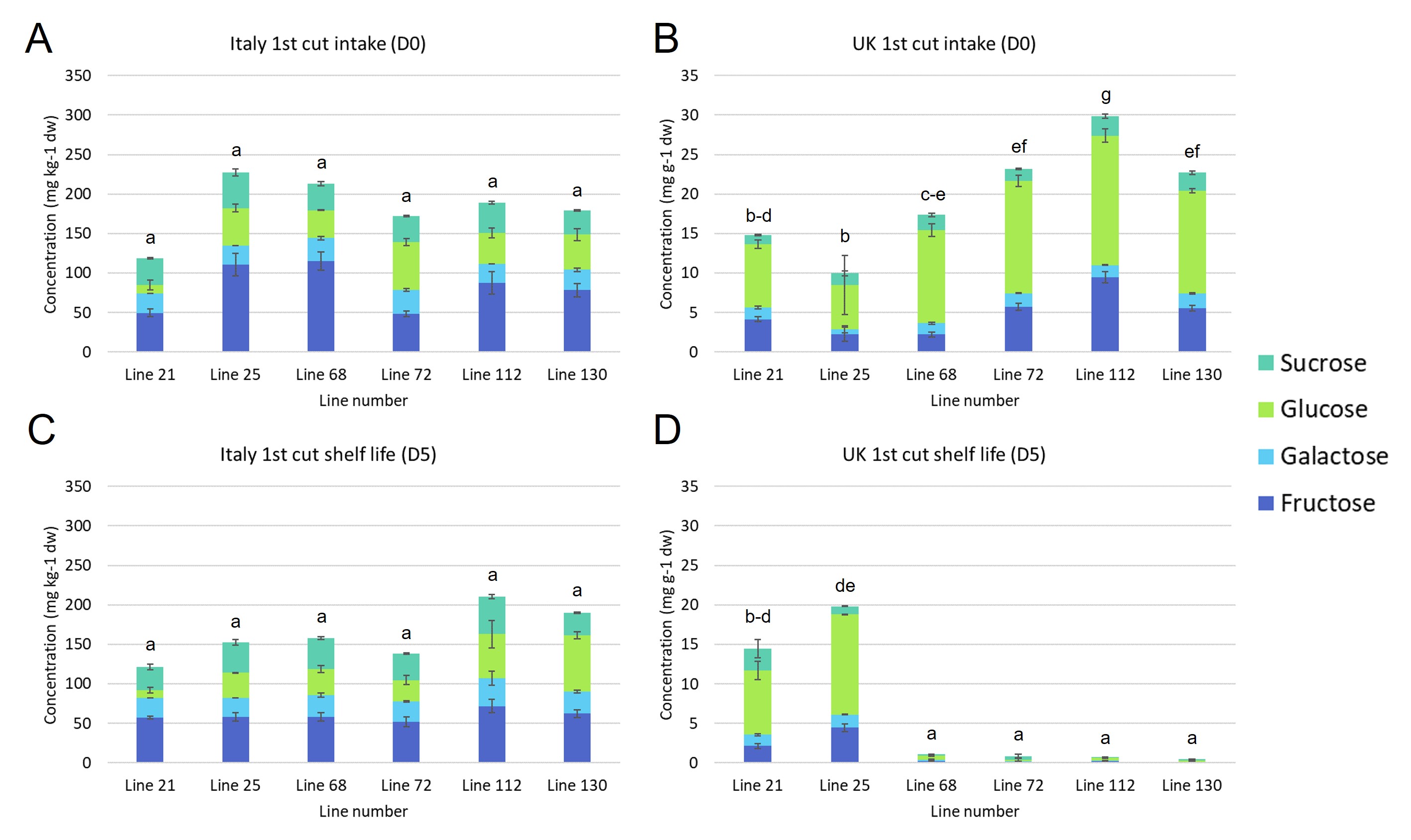

Supplement: Supplementary File S1 — Sampling diagram. [file Image_1.jpeg]
