# Supplementary material for: Genotypes of Eruca vesicaria subsp. sativa grown in contrasting field environments differ on transcriptomic and metabolomic levels, significantly impacting nutritional quality
Source: Front Plant Sci. 2023 Nov 2;14:1218984. doi: 10.3389/fpls.2023.1218984 (PMC10652768; doi:10.3389/fpls.2023.1218984)
Supplement: Supplementary File S2 — List of Volatile Organic Compounds (VOCs) identified in six Eruca vesicaria subsp. sativa extreme lines grown in Italy and the UK. [file Table_1.docx]

| **Trial location** | | **Italy** | | | | **UK** | | | |
| --- | --- | --- | --- | --- | --- | --- | --- | --- | --- |
| **Harvest number** | | 1^st^ cut | | 2^nd^ cut | | 1^st^ cut | | 2^nd^ cut | |
| **No. of bags per cultivar (x6)** | | n = 12 | | n = 12 | | n = 12 | | n = 12 | |
| **Postharvest sampling point / no. of bags, per cultivar** | | D0  n = 6 | D5  n = 6 | D0  n = 6 | D5  n = 6 | D0  n = 6 | D5  n = 6 | D0  n = 6 | D5  n = 6 |
| **No. of bags per analysis type, per cultivar** | **SPME (GC-MS)** | n = 3 | n = 3 | n = 3 | n = 3 | n = 3 | n = 3 | n = 3 | n = 3 |
|  | **RNA & chemical analyses** | n = 3 | n = 3 | n = 3 | n = 3 | n = 3 | n = 3 | n = 3 | n = 3 |
| **Sample preparation method** | **SPME (GC-MS)** | Samples prepared and analysed as fresh leaf material according to Jasper et al. (2020) for day zero (D0) and after five days of storage at 4 °C | | | | | | | |
|  | **RNA & chemical analyses** | Samples frozen at -80 °C on day zero (D0) and after five days of storage at 4 °C. All samples lyophilized for three days. Glucosinolates, hydrolysis products, sugars, sulfur and RNA extracted from dried material. | | | | | | | |
