# Supplementary material for: Genotypes of Eruca vesicaria subsp. sativa grown in contrasting field environments differ on transcriptomic and metabolomic levels, significantly impacting nutritional quality
Source: Front Plant Sci. 2023 Nov 2;14:1218984. doi: 10.3389/fpls.2023.1218984 (PMC10652768; doi:10.3389/fpls.2023.1218984)
Supplement: Supplementary File S3 — Summary table of Analysis of Variance (ANOVA) with post-hoc Tukey’s Honest Significant Difference (HSD) results for Eruca vesicaria subsp. sativa phytochemical compositions. [file Table_2.docx]

| **Table S1.** Volatile organic compounds detected by SPME GC-MS in rocket leaves grown in Italy and the UK | | | | | | | | | | | | | | |
| --- | --- | --- | --- | --- | --- | --- | --- | --- | --- | --- | --- | --- | --- | --- |
| **No** | **Rt** | **Compound identification** | **Compound class** | **Quality score** | **CID number** | **CAS No.** | **LRI ^a^** | **ID ^b^** | **Italy** | **UK** | **Previously observed in** | **Known odour ^c^** | **Known flavour ^c^** | **References** |
| **1** | 2.40 | Ethanol | Alcohol | 90 | 702 | 64-17-5 | 453 | B |  | ✓ | - | Alcoholic, ethereal, medicinal | - | - |
| **2** | 2.64 | Propionaldehyde | Aldehyde | 90 | 527 | 123-38-6 | 496 | A | ✓ | ✓ | Onion | Earthy, nutty | Nutty, vegetative, cheesy, broccoli | ^1^ |
| **3** | 2.81 | (2*E*)-2-pentene | Alkene | 94 | 5326161 | 646-04-8 | 506 | B | ✓ |  | - | - | - | - |
| **4** | 2.90 | Furan | Furan | 91 | 8029 | 110-00-9 | 512 | B |  | ✓ | - | Ethereal | - | - |
| **5** | 2.93 | (2*Z*)-2-pentene | Alkene | 91 | 5326160 | 627-20-3 | 512 | B | ✓ | ✓ | - | - | - | - |
| **6** | 3.02 | Methyl sulfide | Sulfide | 97 | 1068 | 75-18-3 | 517 | B | ✓ | ✓ | Broccoli, Cabbage, Wild rocket, Tomato | Sulfur, onion, vegetable, cabbage, green, radish | Sulfurous, vegetative | ^2–6^ |
| **7** | 3.05 | *Trans*-1,2-dimethylcyclopropane | Cycloalkane | 93 | 6427079 | 2402-06-4 | - | B |  | ✓ | - | - | - | - |
| **8** | 3.10 | (3*E*)-1,3-pentadiene | Alkene | 98 | 62204 | 2004-70-8 | 521 | B | ✓ | ✓ | Fungi | - | - | ^7,8^ |
| **9** | 3.36 | Carbon disulfide | Sulfide | 90 | 6348 | 75-15-0 | 534 | A | ✓ | ✓ | Wild rocket | - | - | ^3,9^ |
| **10** | 5.19 | 3-methylfuran | Furan | 95 | 13587 | 930-27-8 | 612 | A | ✓ | ✓ | Wild rocket, fungi | - | - | ^3,7,9,10^ |
| **11** | 6.09 | Methyl propionate | Carboxylic acid ester | 91 | 11124 | 554-12-1 | 643 | B |  | ✓ | Wild rocket | Fresh, rum, fruity, strawberry, apple | Fruity, ethereal, fruit, rum, banana | ^11^ |
| **12** | 8.47 | <unknown 1> | - | - | - | - | 659 | - |  | ✓ | - | - | - | - |
| **13** | 7.90 | Pent-1-en-3-ol | Alcohol | 91 | 12020 | 616-25-1 | 681 | A | ✓ |  | Tomato | Pungent, horseradish, green, vegetable, fruity | Green, vegetable | ^12^ |
| **14** | 8.56 | Diethyl ketone | Ketone | 83 | 7288 | 96-22-0 | 698 | B | ✓ | ✓ | - | Ether, acetone | - | - |
| **15** | 9.06 | 3-pentanol | Alcohol | 90 | 11428 | 584-02-1 | 701 | B |  | ✓ | - | Sweet, herbal, oily, nutty | - | - |
| **16** | 8.81 | 2-ethylfuran | Furan | 93 | 18554 | 3208-16-0 | 704 | A | ✓ | ✓ | Wild rocket | Sweet, burnt, earthy, malty | Solvent-like, dirty, musty, brown, earthy | ^3,9^ |
| **17** | 9.33 | Methyl thiocyanate | Thiocyanate | 94 | 11168 | 556-64-9 | 714 | B | ✓ | ✓ | - | Sulfur, onion | - | - |
| **18** | 10.08 | 2-methyl-2-pentenal | Aldehyde | 80 | 5319754 | 623-36-9 | 730 | B | ✓ |  | - | Green, grass, fruity, gassy | Sweet, fruity, onion | - |
| **19** | 11.61 | (*E*)-tiglaldehyde | Aldehyde | 81 | 5321950 | 497-03-0 | 753 | B |  | ✓ | Orange | Strong, green, fruit | - | ^13^ |
| **20** | 11.35 | (2*E*)-2-pentenal | Aldehyde | 94 | 5364752 | 764-39-6 | 755 | A | ✓ | ✓ | Orange, Tomato, Wild rocket | Pungent, green, apple, orange, tomato | - | ^13–15^ |
| **21** | 12.42 | (*E*)-2-penten-1-ol | Alcohol | 87 | 5364919 | 1576-96-1 | 770 | B |  | ✓ | - | Mushroom | - | - |
| **22** | 12.09 | (2*Z*)-2-penten-1-ol | Alcohol | 94 | 5364919 | 1576-95-0 | 772 | A | ✓ | ✓ | Cabbage, *Arabidopsis* | Green, plastic, ether, fruity | Mustard, horseradish | ^4,16^ |
| **23** | 12.88 | Methyl 2-methylbutanoate | Fatty acid ester | 90 | 13357 | 868-57-5 | 779 | A |  | ✓ | - | Ethereal, fruity, apple, green, lily, fatty | Ethereal, fruity, apple, green, pear, tropical, floral | - |
| **24** | 13.82 | (*Z*)-3-hexenal | Aldehyde | 94 | 643941 | 6789-80-6 | 799 | A |  | ✓ | - | Green, fatty, grassy, fruity, apple | Sharp, green, grassy, cooked apple, apple | - |
| **25** | 16.04 | 1,1-dimethylcyclopropane | Cycloalkane | 87 | 74202 | 1630-94-0 | 844 | B | ✓ | ✓ | - | - | - | - |
| **26** | 16.40 | 2-ethyl-3-vinyloxirane | Alkane | 91 | 534767 | - | 852 | A |  | ✓ | - | - | - | - |
| **27** | 16.15 | (2*E*)-2-hexenal | Aldehyde | 98 | 5281168 | 6728-26-3 | 854 | A | ✓ | ✓ | Tea, Strawberry, Apple, Arabidopsis, Olive | Green, banana, fatty, cheesy | Fresh, green, leafy, fruity, vegetable | ^17–22^ |
| **28** | 16.43 | (3*Z*)-3-hexen-1-ol | Alcohol | 91 | 5281167 | 544-12-7 | 859 | A | ✓ | ✓ | *Arabidopsis,* Tea, Corn | Green, leafy | - | ^23–25^ |
| **29** | 16.77 | (2*E*)-2-hexen-1-ol | Alcohol | 95 | 5318042 | 928-95-0 | 866 | A | ✓ | ✓ | Olive | Fresh, green, leafy, fruity, unripe banana | Green, leafy, fresh, fatty, grassy, fruity | ^22^ |
| **30** | 17.23 | 1-hexanol | Alcohol | 86 | 8103 | 111-27-3 | 869 | A |  | ✓ | Rapeseed, Olive, Broccoli | Pungent, ethereal, oily, fruity, alcoholic, sweet, green | Green, fruity, apple, oily | ^26–28^ |
| **31** | 18.54 | Methoxy-phenyl-oxime | Oxime | 90 | 9602988 | - | 896 | B |  | ✓ | Broccoli, Brown mustard | - | - | ^29–31^ |
| **32** | 18.34 | (4*Z*)-4-heptenal | Aldehyde | 96 | 5362814 | 6728-31-0 | 899 | A | ✓ |  | Kale | Oily, fatty, green, dairy, milky, creamy | Sharp, green, milky, creamy, dairy, fatty, cheesy, tea | ^32^ |
| **33** | 19.13 | 2-methyl-1,4-hexadiene | Alkene | 80 | 5367524 | 1119-14-8 | 909 | B |  | ✓ | - | - | - | - |
| **34** | 18.91 | (2*E*,4*E*)-2,4-hexadienal | Aldehyde | 90 | 637564 | 142-83-6 | 911 | A | ✓ | ✓ | Wild rocket | Sweet, green, spicy, floral, citrus | Sweet, green, fresh, melon | ^15^ |
| **35** | 19.73 | Methyl hexanoate | Methyl ester | 97 | 7824 | 106-70-7 | 925 | A |  | ✓ | - | Fruity, pineapple, ether | Fruity, fatty | - |
| **36** | 20.08 | Methyl (*Z*)-3-hexenoate | Methyl ester | 92 | 5362819 | 13894-62-7 | 932 | B |  | ✓ | - | Fruity, floral | - | - |
| **37** | 20.55 | 3-ethyl-1,5-octadiene isomer 1 |  | 80 | 5353002 |  | 940 | B | ✓ |  | *Arabidopsis* | - | - | ^16^ |
| **38** | 20.49 | 5-methylhexanenitrile | Nitrile | 83 | 29593 | 19424-34-1 | 946 | B | ✓ |  | Salad rocket, Turnip | - | - | ^33,34^ |
| **39** | 20.56 | 3-ethyl-1,5-octadiene isomer 2 | Alkene | 86 | 5353002 | - | 947 | B | ✓ | ✓ | *Arabidopsis* | - | - | ^16^ |
| **40** | 20.93 | 6-methylheptan-2-one | Ketone | 83 | 13572 | 928-68-7 | 956 | B | ✓ |  | - | Camphor | - | - |
| **41** | 21.02 | (*E*)-4-oxohex-2-enal | Aldehyde | 91 | 6365145 | 2492-43-5 | 958 | B | ✓ | ✓ | Wild rocket | - | - | ^15^ |
| **42** | 21.34 | (4*Z*)-4-hepten-1-ol | Alcohol | 83 | 5367536 | 20851-55-2 | 965 | B | ✓ |  | - | - | - | - |
| **43** | 21.78 | Benzaldehyde | Benzene aldehyde | 96 | 240 | 100-52-7 | 967 | B |  | ✓ | Rapeseed | Strong, sharp, sweet, almond, cherry | Sweet, oily, almond, cherry, nutty, woody | ^26^ |
| **44** | 22.02 | Heptanenitrile | Nitrile | 93 | 12372 | 629-08-3 | 980 | A | ✓ |  | - | - | - | - |
| **45** | 22.52 | 2,3-octanedione | Ketone | 83 | 11449 | 585-25-1 | 984 | B |  | ✓ | Horseradish | Dill, asparagus, corriander, herbal, earthy, fatty | Green, spicy, corriander, fatty, leafy, herbal | ^35^ |
| **46** | 22.33 | Methyl heptanone | Ketone | 92 | 9862 | 110-93-0 | 987 | A | ✓ |  | Radish | Citrus, green, musty, lemongrass, apple | Green, vegetative, musty, apple, banana, green, bean-like | ^36^ |
| **47** | 22.60 | Myrcene | Monoterpene | 90 | 31253 | 123-35-3 | 992 | A | ✓ |  | *Arabidopsis* | Peppery, spicy, plastic | Woody, vegetative, citrus, fruity, mango, leafy, minty | ^16^ |
| **48** | 23.00 | 2-pentylfuran | Furan | 93 | 19602 | 3777-69-3 | 994 | A |  | ✓ | Olive, Tomato, Radiccio, Lettuce, Broccoli, Cantaloupe, Rice | Fruity, green, earthy, beany, vegetative, metallic | Fruity, green, earthy, beany, vegetative | ^5,29,37–41^ |
| **49** | 22.82 | 2,4-heptadienal isomer 1 | Aldehyde | 97 | 5283321 | 4313-03-5 | 997 | A | ✓ | ✓ | Anchovy | Fatty, green, oily, vegetable, cinnamon | Fatty, green | ^42^ |
| **50** | 22.94 | <unknown isothiocyanate 1> | Isothiocyanate | - | - | - | 1000 | - | ✓ |  | - | - | - | - |
| **51** | 23.44 | (*Z*)-2-(2-pentenyl)furan | Furan | 90 | 5370006 | 70424-13-4 | 1004 | B |  | ✓ | - | - | - | - |
| **52** | 23.16 | (3*Z*)-3-hexenyl acetate | Alkenyl ester | 90 | 5363388 | 3681-71-8 | 1005 | A | ✓ |  | Curuba, Olive, Kale, Arabidopsis, Broccoli, Lettuce, Cabbage, Wild cabbage, Black mustard | Fresh, green, sweet, fruity, banana, apple, grassy | Green, fruity, apple, pear, fresh | ^16,22,28,43–47^ |
| **53** | 23.56 | Hex-4-enyl acetate | Acetate ester | 90 | 529513 | 42125-17-7 | 1007 | B |  | ✓ | - | - | - | - |
| **54** | 23.81 | Hexyl acetate | Acetate ester | 90 | 8908 | 142-92-7 | 1013 | B |  | ✓ | Olive | Fruity, green, apple, banana, sweet | Fruity, green, fresh, sweet, banana, apple, pear | ^27^ |
| **55** | 23.44 | 2,4-heptadienal isomer 2 | Aldehyde | 95 | 5283321 | 4313-03-5 | 1014 | A | ✓ | ✓ | Anchovy | Fatty, green, oily, vegetable, cinnamon | Fatty, green | ^42^ |
| **56** | 23.52 | 2-hexenyl acetate | Alkenyl ester | 90 | 5363374 | 10094-40-3 | 1014 | B | ✓ |  | - | Green, fruity | - | - |
| **57** | 24.37 | <unknown 2> | - | - | - | - | 1026 | - |  | ✓ | - | - | - | - |
| **58** | 24.14 | 2-ethylhexanol | Alcohol | 86 | 7720 | 104-76-7 | 1028 | A | ✓ |  | Fungi, Apricot, Plum | Citrus, fresh, floral, oily, sweet | Sweet, fatty, fruity | ^7,48^ |
| **59** | 24.44 | (+)-limonene | Monoterpene | 98 | 440917 | 138-86-3 | 1036 | A | ✓ |  | Kale | Citrus, herbal, camphor | - | ^44^ |
| **60** | 25.10 | 2,2,6-trimethylcyclohexanone | Cyclohexyl ketone | 96 | 17000 | 2408-37-9 | 1043 | B |  | ✓ | Apricot, Plum, Kale, Turnip | Pungent, thujonic, honey | - | ^32,48,49^ |
| **61** | 25.01 | Phenylacetaldehyde | Aldehyde | 94 | 998 | 122-78-1 | 1049 | A | ✓ |  | Salad rocket | Green, sweet, floral, hyacinth, clover, honey, cocoa | Honey, sweet, floral, chocolate, cocoa, spicy | ^33^ |
| **62** | 25.77 | 1-iodo-2-methylnonane | Halogenated alkane | 93 | 537317 | - | 1059 | B |  | ✓ | - | - | - | - |
| **63** | 25.63 | Isoamyl isothiocyanate | Isothiocyanate | 90 | 79086 | 628-03-5 | 1064 | B | ✓ | ✓ | Salad rocket | Green | - | ^50^ |
| **64** | 25.98 | (*E,Z*)-1,3,5-octatriene | Alkene | 89 | 5367475 | 33580-05-1 | 1072 | B | ✓ |  | - | Green, plastic | - | - |
| **65** | 26.17 | 3-methyldecane | Alkane | 93 | 92239 | 13151-34-3 | 1072 | B |  | ✓ | - | - | - | - |
| **66** | 27.35 | Undecane | Alkane | 91 | 14257 | 1120-21-4 | 1097 | B |  | ✓ | Radish, Broccoli, Coffee, Salad rocket, Onion | - | - | ^29,51–55^ |
| **67** | 27.04 | 2-isopropyl-3-methoxypyrazine | Pyrazine | 86 | 33166 | 25773-40-4 | 1098 | B | ✓ |  | White mustard, Rapeseed | Peas, earthy, beany, chocolate, nutty | - | ^56^ |
| **68** | 27.12 | <unknown 3> | - | - | - | - | 1099 | - | ✓ |  | - | - | - | - |
| **69** | 27.24 | Amyl isothiocyanate | Isothiocyanate | 86 | 69414 | 629-12-9 | 1103 | B | ✓ | ✓ | - | Green | - | - |
| **70** | 27.61 | 2-benzylidenehydrazono-3-methyl-2,3-dihydrobenzothiazole | Benzyl thiazole | 94 | 5735562 | - | 1103 | B |  | ✓ | - | - | - | - |
| **71** | 27.34 | Nonanal | Aldehyde | 96 | 31289 | 124-19-6 | 1105 | A | ✓ | ✓ | Olive, Broccoli, Rice | Waxy, rose, fresh, orange peel, fatty | Citrus, cucumber, melon, raw potato, oily, nutty, coconut | ^37,41,57^ |
| **72** | 27.83 | 2,6-dimethylcyclohexanol | Alcohol | 87 | 21428 | 5337-72-4 | 1117 | B | ✓ | ✓ | Apricot, Plum, Wallflower | - | - | ^48,58^ |
| **73** | 28.30 | Methyl octanoate | Fatty acid methyl ester | 87 | 8091 | 111-11-5 | 1121 | B |  | ✓ | Papaya | Waxy, green, sweet, orange, vegetable, herbal | Green, fruity, waxy, citrus, fatty | ^59^ |
| **74** | 29.09 | <unknown 4> | - | - | - | - | 1141 | - |  | ✓ | - | - | - | - |
| **75** | 29.31 | Tetrahydrothiophene | Thiophene | 91 | 1127 | 110-01-0 | 1155 | B | ✓ | ✓ | Salad rocket | Cabbage | - | ^60^ |
| **76** | 29.98 | <unknown 5> | - | - | - | - | 1164 | - |  | ✓ | - | - | - | - |
| **77** | 29.83 | 1-isothiocyanato-4-methylpentane | Isothiocyanate | 95 | 519452 | 17608-07-0 | 1168 | A | ✓ | ✓ | - | - | - | - |
| **78** | 30.15 | 2-sec-butyl-3-methoxypyrazine | Pyrazine | 91 | 520098 | 24168-70-5 | 1177 | A | ✓ | ✓ | - | Musty, green, peas, bell pepper | Musty, vegetative, potato, fishy, earthy | - |
| **79** | 30.49 | 2-isobutyl-3-methoxypyrazine | Pyrazine | 95 | 32594 | 24683-00-9 | 1185 | A | ✓ |  | White mustard, Rapeseed, Red wine | Green, peas, bell pepper | Green, bell pepper, peas | ^56,61^ |
| **80** | 30.75 | (*E*)-butanoic acid 3-hexenyl ester | Methyl ester | 83 | 5352331 | 53398-84-8 | 1185 | A |  | ✓ |  | - | - |  |
| **81** | 30.51 | (3*Z*)-3-hexenyl butyrate | Alkenyl | 86 | 5352438 | 16491-36-4 | 1186 | B | ✓ |  | Brussels sprouts, Salad rocket | Fresh, green, apple, fruity, metallic, buttery | Fresh, green, apple, pear, vegetative | ^62,63^ |
| **82** | 30.74 | Dodecene | Alkene | 97 | 8183 | 112-41-4 | 1191 | A | ✓ |  | Lettuce, Cabbage | - | - | ^45^ |
| **83** | 31.29 | <unknown isothiocyanate 2> | Isothiocyanate | - | - | - | 1206 | - | ✓ | ✓ | - | - | - | - |
| **84** | 31.32 | 5-(methylsulfanyl)pentanenitrile | Nitrile | 91 | 93320 | 59121-25-4 | 1207 | B | ✓ | ✓ | Swede, Turnip | Broccoli, cabbage | - | ^34,64^ |
| **85** | 32.28 | β-cyclocitral | Monoterpene | 99 | 9895 | 432-25-7 | 1235 | A | ✓ | ✓ | *Arabidopsis,* Tomato, ﻿Aurinia, Brown mustard, Kale, Rapeseed, Spanish cistus, Apricot, Plum, Salad rocket, Tronchuda cabbage | Tropical, saffron, herbal, rose, sweet, tobacco, fruity | Tropical, saffron, herbal, tobacco, medicinal, leather, green | ^12,16,32,33,44,48,49,65–68^ |
| **86** | 35.57 | Iberverin | Isothiocyanate | 91 | 62351 | 505-79-3 | 1324 | B | ✓ |  | - | Earthy, vegetable, sulfurous, horseradish | Vegetative, radish, sulfurous, earthy, metallic | - |
| **87** | 32.80 | <unknown 6> | - | - | - | - | 1238 | - |  | ✓ | *-* | - | - | - |
| **88** | 34.10 | β-homocyclocitral | Monoterpene | 96 | 61124 | 472-66-2 | 1274 | B |  | ✓ | Kale, Tronchuda kale, Turnip | Camphoreous, cooling, woody, fruity | Cooling, woody, oily, soapy, citrus, berry | ^32,49,68^ |
| **89** | 36.04 | *Cis*-3-hexenyl 2-methylbutenoate | Fatty acid ester | 80 | 5365069 | - | 1328 | B |  | ✓ | *-* | Fresh, green, herbal, apple, sweet, fruity, pineapple | Green, fruity, pineapple, pear, apple, melon | - |
| **90** | 36.78 | Sativin | Thiazepane-2-thione | - | - | - | 1359 | A | ✓ | ✓ | Salad rocket | Rocket-like | - | ^69^ |
| **91** | 37.90 | Tetradecene | Alkene | 99 | 14260 | 26952-13-6 | 1392 | A |  | ✓ | - | - | - | - |
| **92** | 39.73 | Erucin | Isothiocyanate | - | 78160 | 4430-36-8 | 1448 | A | ✓ | ✓ | Salad rocket | Cabbage, radish | - | ^70^ |
| **93** | 41.32 | <unknown isothiocyanate 3> | Isothiocyanate | - | - | - | 1487 | - |  | ✓ | - | - | - | - |
| **94** | 41.43 | β-ionone | Monoterpene | 97 | 638014 | 14901-07-6 | 1501 | A | ✓ | ✓ | Tomato, Tea | Floral, woody, sweet, fruity, berries, tropical | Woody, berries, floral, green, fruity | ^5,14,71,72^ |
| **95** | 48.20 | 2-tridecyloxirane | Cycloalkane | 96 | 86768 | - | 1719 | B |  | ✓ | - | - | - | - |
| **96** | 53.72 | Methyl palmitate | Fatty acid ester | 97 | 8181 | 112-39-0 | 1927 | B |  | ✓ | *Erysimum corinthium*, Chinese cabbage, Rice, Radish | Oily, waxy, fatty | - | ^41,52,58,73^ |
| ^a^ = Linear retention index on a HP-5MS column. ^b^ = A, mass spectrum and LRI agree with those of authentic compound; B, mass spectrum agrees with reference spectrum in the NIST/EPA/NIH mass spectra database and LRI agree with those in the literature; tentatively identified. ^c^ = Odour description of compound present in the Good Scents online database: http://www.thegoodscentscompany.com/(accessed on 1 April 2022) and literature sources. | | | | | | | | | | | | | | |

**References**

1 Virtanen AI. Studies on organic sulphur compounds and other labile substances in plants. *Phytochemistry* 1965; **4**: 207–228.

2 Spadone J-C, Matthey-Doret W, Blank I. Formation of methyl (methylthio)methyl disulfide in broccoli (*Brassica oleracea* (L.) var. *italica*). *Dev Food Sci* 2006; **43**: 309–314.

3 Luca A, Mahajan PV, Edelenbos M. Changes in volatile organic compounds from wild rocket (*Diplotaxis tenuifolia* L.) during modified atmosphere storage. *Postharvest Biol Technol* 2016; **114**: 1–9.

4 Rajkumar G, Shanmugam S, Galvâo M de S *et al.* Comparative evaluation of physical properties and volatiles profile of cabbages subjected to hot air and freeze drying. *LWT* 2017; **80**: 501–509.

5 Buttery RG, Teranishi R, Ling LC, Turnbaugh JG. Quantitative and Sensory Studies on Tomato Paste Volatiles. *J Agric Food Chem* 1990; **38**: 336–340.

6 Chin H-W, Lindsay RC. Volatile Sulfur Compounds Formed in Disrupted Tissues of Different Cabbage Cultivars. *J Food Sci* 1993; **58**: 835–839.

7 Sunesson A, Vaes W, Nilsson C, Blomquist G, Andersson B, Carlson R. Identification of volatile metabolites from five fungal species cultivated on two media. *Appl Environ Microbiol* 1995; **61**: 2911–2918.

8 Huber SG, Wunderlich S, Schöler HF, Williams J. Natural Abiotic Formation of Furans in Soil. *Environ Sci Technol* 2010; **44**: 5799–5804.

9 Luca A, Kjaer A, Edelenbos M. Volatile organic compounds as markers of quality changes during the storage of wild rocket. *Food Chem* 2017; **232**: 597–586.

10 Börjesson T, Stöllman U, Schnürer J. Volatile metabolites produced by six fungal species compared with other indicators of fungal growth on cereal grains. *Appl Environ Microbiol* 1992; **58**: 2599–2605.

11 Caruso G, Parrella G, Giorgini M, Nicoletti R. Crop Systems, Quality and Protection of *Diplotaxis tenuifolia*. *Collect FAO Agric* 2018; **8**: 55–55.

12 Wang C, Xing J, Chin C-K, Ho C-T, Martin CE. Modification of fatty acids changes the flavor volatiles in tomato leaves. *Phytochemistry* 2001; **58**: 227–232.

13 Moshonas MG, Shaw PE. Some Newly Found Orange Essence Components Including *Trans*-2-Pentenal. *J Food Sci* 1973; **38**: 360–361.

14 Tandon KS, Baldwin EA, Shewfelt RL. Aroma perception of individual volatile compounds in fresh tomatoes (*Lycopersicon esculentum*, Mill.) as affected by the medium of evaluation. *Postharvest Biol Technol* 2000; **20**: 261–268.

15 Spadafora ND, Amaro AL, Pereira MJ, Müller CT, Pintado M, Rogers HJ. Multi-trait analysis of post-harvest storage in rocket salad (*Diplotaxis tenuifolia*) links sensorial, volatile and nutritional data. *Food Chem* 2016; **211**: 114–123.

16 Rohloff J, Bones AM. Volatile profiling of *Arabidopsis thaliana* - Putative olfactory compounds in plant communication. *Phytochemistry* 2005; **66**: 1941–1955.

17 Hatanaka A, Harada T. Formation of *Cis*-3-Hexenal, *Trans*-2-Hexenal and *Cis*-3-Hexenol in Macerated *Thea sinensis* Leaves. *Phytochemistry* 1973; **12**: 2341–2346.

18 Corbo MR, Lanciotti R, Gardini F, Sinigaglia M, Guerzoni ME. Effects of hexanal, *trans*-2-hexenal, and storage temperature on shelf life of fresh sliced apples. *J Agric Food Chem* 2000; **48**: 2401–2408.

19 Fallik E, Archbold DD, Hamilton-Kemp TR, Clements AM, Collins RW, Barth MM. (*E*)-2-hexenal can stimulate *Botrytis cinerea* growth *in vitro* and on strawberries *in vivo* during storage. *J Am Soc Hortic Sci* 1998; **123**: 875–881.

20 Hatanaka A, Kajiwara T, Sekiya J. Biosynthesis of *Trans*-2-Hexenal in Chloroplasts from *Thea sinensis*. *Phytochemistry* 1976; **15**: 1125–1126.

21 Mirabella R, Rauwerda H, Struys EA *et al.* The Arabidopsis her1 mutant implicates GABA in *E*-2-hexenal responsiveness. *Plant J* 2008; **53**: 197–213.

22 Angerosa F. Virgin olive oil odour notes: their relationships with volatile compounds from the lipoxygenase pathway and secoiridoid compounds. *Food Chem* 2000; **68**: 283–287.

23 D’Auria JC, Pichersky E, Schaub A, Hansel A, Gershenzon J. Characterization of a BAHD acyltransferase responsible for producing the green leaf volatile (*Z*)-3-hexen-1-yl acetate in *Arabidopsis thaliana*. *Plant J* 2007; **49**: 194–207.

24 Saijo R, Takeo T. Increase of *Cis*-3-Hexen-1-Ol Content in Tea Leaves Following Mechanical Injury. *Phytochemistry* 1975; **14**: 181–182.

25 Ruther J, Kleier S. Plant-plant signaling: Ethylene synergizes volatile emission in *Zea mays* induced by exposure to (*Z*)-3-Hexen-1-ol. *J Chem Ecol* 2005; **31**: 2217–2222.

26 Bartlet E, Blight MM, Lane P, Williams IH. The responses of the cabbage seed weevil *Ceutorhynchus assimilis* to volatile compounds from oilseed rape in a linear track olfactometer. *Entomol Exp Appl* 1997; **85**: 257–262.

27 Servili M, Selvaggini R, Taticchi A, Esposto S, Montedoro GF. Volatile compounds and phenolic composition of virgin olive oil: Optimization of temperature and time of exposure of olive pastes to air contact during the mechanical extraction process. *J Agric Food Chem* 2003; **51**: 7980–7988.

28 Koutidou M, Grauwet T, Van Loey A, Acharya P. Potential of different mechanical and thermal treatments to control off-flavour generation in broccoli puree. *Food Chem* 2017; **217**: 531–541.

29 Deasy W, Shepherd T, Alexander CJ, Birch ANE, Evans KA. Development and Validation of a SPME-GC-MS Method for *In situ* Passive Sampling of Root Volatiles from Glasshouse-Grown Broccoli Plants Undergoing Below-Ground Herbivory by Larvae of Cabbage Root Fly, *Delia radicum* L. *Phytochem Anal* 2016; **27**: 375–393.

30 Luo F, Cai J-H, Zhang X *et al.* Effects of methyl jasmonate and melatonin treatments on the sensory quality and bioactive compounds of harvested broccoli. *RSC Adv* 2018; **8**: 41422–41431.

31 Sharma A, Rai PK, Prasad S. GC–MS detection and determination of major volatile compounds in *Brassica juncea* L. leaves and seeds. *Microchem J* 2018; **138**: 488–493.

32 Fernandes F, Guedes de Pinho P, Valentão P, Pereira JA, Andrade PB. Volatile constituents throughout *Brassica oleracea* L. Var. *acephala* germination. *J Agric Food Chem* 2009; **57**: 6795–6802.

33 Blažević I, Mastelić J. Free and bound volatiles of rocket (*Eruca sativa* Mill.). *Flavour Fragr J* 2008; **23**: 278–285.

34 Tomita S, Nakamura T, Okada S. NMR- and GC/MS-based metabolomic characterization of sunki, an unsalted fermented pickle of turnip leaves. *Food Chem* 2018; **258**: 25–34.

35 Dekic MS, Radulovic NS, Stojanovic NM *et al.* Spasmolytic, antimicrobial and cytotoxic activities of 5-phenylpentyl isothiocyanate, a new glucosinolate autolysis product from horseradish (*Armoracia rusticana* P. Gaertn., B. Mey. & Scherb., Brassicaceae). *Food Chem* 2017; **232**: 329–339.

36 Chen W, Karangwa E, Yu J *et al.* Characterizing Red Radish Pigment Off-Odor and Aroma-Active Compounds by Sensory Evaluation, Gas Chromatography-Mass Spectrometry/Olfactometry and Partial Least Square Regression. *Food Bioprocess Technol* 2017; **10**: 1–17.

37 Vichi S, Pizzale L, Conte LS, Buxaderas S, López-Tamames E. Solid-Phase Microextraction in the Analysis of Virgin Olive Oil Volatile Fraction: Modifications Induced by Oxidation and Suitable Markers of Oxidative Status. *J Agric Food Chem* 2003; **51**: 6564–6571.

38 Cozzolino R, Martignetti A, Pellicano MP *et al.* Characterisation of volatile profile and sensory analysis of fresh-cut “Radicchio di Chioggia” stored in air or modified atmosphere. *Food Chem* 2016; **192**: 603–611.

39 Deza-Durand KM, Petersen MA. Volatile compounds of modified atmosphere packaged cut iceberg lettuce: Effect of extremely low O2, season, cultivar and storage time. *Food Res Int* 2014; **62**: 254–261.

40 Beaulieu JC, Grimm CC. Identification of Volatile Compounds in Cantaloupe at Various Developmental Stages Using Solid Phase Microextraction. *J Agric Food Chem* 2001; **49**: 1345–1352.

41 Buttery RG, Turnbaugh JG, Ling LC. Contribution of Volatiles to Rice Aroma. *J Agric Food Chem* 1988; **36**: 1006–1009.

42 Triqui R, Reineccius GA. Changes in flavor profiles with ripening of anchovy (*Engraulis encrasicholus*). *J Agric Food Chem* 1995; **43**: 1883–1889.

43 Conde-Martínez N, Sinuco DC, Osorio C. Chemical studies on curuba (*Passiflora mollissima* (Kunth) L. H. Bailey) fruit flavour. *Food Chem* 2014; **157**: 356–363.

44 Fernandes F, Pereira DM, Guedes de Pinho P *et al.* Headspace solid-phase microextraction and gas chromatography/ion trap-mass spectrometry applied to a living system: *Pieris brassicae* fed with kale. *Food Chem* 2010; **119**: 1681–1693.

45 Lonchamp J, Barry-Ryan C, Devereux M. Identification of volatile quality markers of ready-to-use lettuce and cabbage. *Food Res Int* 2009; **42**: 1077–1086.

46 Breme K, Langle S, Fernandez X, Meierhenrich UJ, Brevard H, Joulain D. Character impact odorants from Brassicaceae by aroma extract dilution analysis (AEDA): *Brassica cretica* and *Brassica insularis*. *Flavour Fragr J* 2009; **24**: 88–93.

47 Kask K, Kännaste A, Talts E, Copolovici L, Niinemets Ü. How specialized volatiles respond to chronic and short-term physiological and shock heat stress in *Brassica nigra*. *Plant Cell Environ* 2016; **39**: 2027–2042.

48 Gomez E, Ledbetter C, Hartsell PL. Volatile compounds in apricot, plum and their interspecific hybrids. *J Agric Food Chem* 1993; **41**: 1669–1676.

49 Taveira M, Fernandes F, Guedes de Pinho P, Andrade PB, Pereira JA, Valentão P. Evolution of Brassica rapa var. rapa L. volatile composition by HS-SPME and GC/IT-MS. *Microchem J* 2009; **93**: 140–146.

50 Bell L, Spadafora ND, Müller CT, Wagstaff C, Rogers HJ. Use of TD-GC-TOF-MS to assess volatile composition during post-harvest storage in seven accessions of rocket salad (*Eruca sativa*). *Food Chem* 2016; **194**: 626–636.

51 Beevi SS, Mangamoori LN, Subathra M, Edula JR. Hexane Extract of *Raphanus sativus* L. Roots Inhibits Cell Proliferation and Induces Apoptosis in Human Cancer Cells by Modulating Genes Related to Apoptotic Pathway. *Plant Foods Hum Nutr* 2010; **65**: 200–209.

52 Blažević I, Mastelić J. Glucosinolate degradation products and other bound and free volatiles in the leaves and roots of radish (*Raphanus sativus* L.). *Food Chem* 2009; **113**: 96–102.

53 Chen H-Z, Zhang M, Guo Z. Discrimination of fresh-cut broccoli freshness by volatiles using electronic nose and gas chromatography-mass spectrometry. *Postharvest Biol Technol* 2019; **148**: 168–175.

54 Miyazawa M, Maehara T, Kurose K. Composition of the essential oil from the leaves of *Eruca sativa*. *Flavour Fragr J* 2002; **17**: 187–190.

55 Wang A, Luca A, Edelenbos M. Emission of volatile organic compounds from yellow onion (*Allium cepa* L.) bulbs during storage. *J Food Sci Technol* 2019; : 1–9.

56 Ortner E, Granvogl M, Schieberle P. Elucidation of Thermally Induced Changes in Key Odorants of White Mustard Seeds (*Sinapis alba* L.) and Rapeseeds (*Brassica napus* L.) Using Molecular Sensory Science. *J Agric Food Chem* 2016; **64**: 8179–8190.

57 Hansen M, Laustsen AM, Olsen CE, Poll L, Sorensen H. Chemical and sensory quality of broccoli (*Brassica oleracea* L. var *italica*). *J Food Qual* 1997; **20**: 441–459.

58 Al-Gendy AA, El-gindi OD, Hafez AS, Ateya AM. Glucosinolates, volatile constituents and biological activities of *Erysimum corinthium* Boiss. (Brassicaceae). *Food Chem* 2010; **118**: 519–524.

59 Lieb VM, Esquivel P, Cubero Castillo E, Carle R, Steingass CB. GC-MS profiling, descriptive sensory analysis, and consumer acceptance of Costa Rican papaya (*Carica papaya* L.) fruit purees. *Food Chem* 2018; **248**: 238–246.

60 Bell L, Methven L, Signore A, Jose Oruna-Concha M, Wagstaff C. Analysis of Seven Salad Rocket (*Eruca sativa*) Accessions: The Relationships Between Sensory Attributes and Volatile and Non-volatile Compounds. *Food Chem* 2017; **218**: 181–191.

61 Frank S, Wollmann N, Schieberle P, Hofmann T. Reconstitution of the Flavor Signature of Dornfelder Red Wine on the Basis of the Natural Concentrations of Its Key Aroma and Taste Compounds. *J Agric Food Chem* 2011; **59**: 8866–8874.

62 Smid HM, van Loon JJA, Posthumus MA, Vet LEM. GC-EAG-analysis of volatiles from Brussels sprouts plants damaged by two species of *Pieris* caterpillars: olfactory receptive range of a specialist and a generalist parasitoid wasp species. *Chemoecology* 2002; **12**: 169–176.

63 Jirovetz L, Smith D, Buchbauer G. Aroma compound analysis of *Eruca sativa* (Brassicaceae) SPME headspace leaf samples using GC, GC-MS, and olfactometry. *J Agric Food Chem* 2002; **50**: 4643–4646.

64 Klopsch R, Witzel K, Börner A, Schreiner M, Hanschen FS. Metabolic profiling of glucosinolates and their hydrolysis products in a germplasm collection of *Brassica rapa* turnips. *Food Res Int* 2017; **100**: 392–403.

65 Blažević I, Radonić A, Mastelić J, Zekić M, Skočibušić M, Maravić A. Glucosinolates, glycosidically bound volatiles and antimicrobial activity of *Aurinia sinuata* (Brassicaceae). *Food Chem* 2010; **121**: 1020–1028.

66 Zhao DY, Tang J, Ding XL. Analysis of volatile components during potherb mustard (*Brassica juncea*, Coss.) pickle fermentation using SPME-GC-MS. *Lwt-Food Science and Technology* 2007; **40**: 439–447.

67 Morales-Soto A, Oruna-Concha MJ, Elmore JS *et al.* Volatile profile of Spanish Cistus plants as sources of antimicrobials for industrial applications. *Ind Crops Prod* 2015; **74**: 425–433.

68 de Pinho PG, Valentão P, Gonçalves RF, Sousa C, Andrade PB. Volatile composition of *Brassica oleracea* L. var. *costata* DC leaves using solid-phase microextraction and gas chromatography/ion trap mass spectrometry. *Rapid Commun Mass Spectrom* 2009; **23**: 2292–2300.

69 Fechner J, Kaufmann M, Herz C *et al.* The major glucosinolate hydrolysis product in rocket (*Eruca sativa* L.), sativin, is 1,3-thiazepane-2-thione: Elucidation of structure, bioactivity, and stability compared to other rocket isothiocyanates. *Food Chem* 2018; **261**: 57–65.

70 Raffo A, Masci M, Moneta E *et al.* Characterization of volatiles and identification of odor-active compounds of rocket leaves. *Food Chem* 2018; **240**: 1161–1170.

71 Baldwin EA, Scott JW, Einstein MA *et al.* Relationship between sensory and instrumental analysis for tomato flavor. *J Am Soc Hortic Sci* 1998; **123**: 906–915.

72 Gong X, Han Y, Zhu J *et al.* Identification of the aroma-active compounds in Longjing tea characterized by odor activity value, gas chromatography-olfactometry, and aroma recombination. *Int J Food Prop* 2017; **20**: S1107–S1121.

73 Daxenbichler ME, Vanetten CH, Williams PH. Glucosinolates and derived products in cruciferous vegetables - analysis of 14 varieties of chinese cabbage. *J Agric Food Chem* 1979; **27**: 34–37.
